# Predictive Value of Diagnostic Methods for TMJ Hypermobility in the Associated Clinical and Functional Features of Temporomandibular Disorders: A Regression Study

**DOI:** 10.1111/joor.70120

**Published:** 2025-11-27

**Authors:** Samilla Pontes Braga, Dyanne Medina Flores, Maria Emilia Servin Berden, Ana Claudia de Castro Ferreira Conti, Ambrosina Michelotti, Paulo César Rodrigues Conti

**Affiliations:** ^1^ Bauru Orofacial Pain Group, Department of Prosthodontics and Periodontology, Bauru School of Dentistry University of São Paulo Bauru São Paulo Brazil; ^2^ Bauru Orofacial Pain Group, Department of Orthodontics, Bauru School of Dentistry University of Sao Paulo Bauru Sao Paulo Brazil; ^3^ Postgraduate Program in Orthodontics and Master Program in TMD/Orofacial Pain, Faculty of Dentistry University of Naples Federico II Naples Italy

**Keywords:** articular, joint dislocations, joint instability, orofacial pain, range of motion, temporomandibular joint, temporomandibular joint disorders

## Abstract

**Background:**

Joint hypermobility (JH), particularly at the temporomandibular joint (TMJ), has been proposed as a potential risk factor for temporomandibular disorders (TMD). However, its heterogeneous diagnostic approaches (clinical, anamnestic and imaging) limit the identification of reliable predictors of masticatory dysfunction and TMD outcomes.

**Objectives:**

To determine which diagnostic methods for TMJ hypermobility best predict masticatory dysfunction and the clinical and functional repercussions of TMD.

**Methods:**

This cross‐sectional study included 126 adults recruited at the Bauru School of Dentistry. Participants were classified by Diagnostic Criteria for TMD (DC/TMD) into painful, dysfunction or combined groups, balanced by history of open‐locking. Assessments comprised DC/TMD and ICOP examinations, symptom intensity on the visual analog scale (VAS), Beighton score, pressure pain thresholds, bite‐force and fatigue tests, ultrasound of the TMJ capsule and masseter, CBCT‐based condylar angle and validated measures of mandibular function and psychosocial factors.

**Results:**

Bivariate analyses yielded associations, but multivariate models identified specific predictors. Open‐locking independently predicted TMJ subluxation and higher joint instability. Increased condylar angle was associated with greater assisted opening and midline deviation. Unassisted opening predicted a larger condylar angle, greater pain‐free opening and lateral condylar jump; assisted opening similarly predicted pain‐free opening and lateral condylar jump. Lateral condylar jump independently predicted functional limitation in wide‐opening tasks.

**Conclusion:**

Clinical, functional and imaging indicators of TMJ hypermobility capture distinct aspects of the condition. While open‐locking, condylar angle and mouth‐opening measures predict specific features, no single marker explains the full spectrum and TMJ hypermobility shows limited impact on overall TMD outcomes.

## Graphical abstract

Graphical abstract illustrating diagnostic methods of TMJ hypermobility and their predictive value for masticatory dysfunction and TMD outcomes.

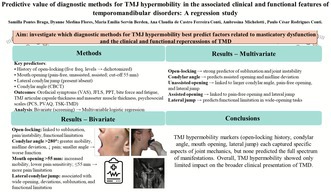

## 1 Background

Joint hypermobility (JH) refers to the excessive movement of a joint in its normal plane. When it affects multiple joints, primarily the limbs and axial skeleton, it is called generalised joint hypermobility (GJH). Temporomandibular joint (TMJ) hypermobility, in turn, leads to movement beyond normal during condylar translation, when the condyle passes the crest of the articular eminence. Joint hypermobility (generalised and TMJ localised) has long been debated as a potential risk factor for temporomandibular disorders (TMD) [1]. While TMJ hypermobility may present asymptomatically in many individuals, in some cases it progresses to clinically significant subluxations, luxations or ‘open‐lock’ episodes that compromise orofacial function [2]. GJH has been hypothesised as a systemic factor contributing to TMJ hypermobility and TMD [3, 4].

A recent scoping review highlighted that while several studies reported a higher prevalence of intra‐articular TMD in hypermobile individuals, others found no correlation. Importantly, TMJ hypermobility, often referred to interchangeably as TMJ hypertranslation or subluxation, has been inconsistently diagnosed using clinical measures (e.g., maximum mouth opening), ‘open‐lock’ episodes history (condition in which the mandible becomes stuck in maximal or near‐maximal opening, and the patient is unable to close it spontaneously until manual or spontaneous reduction of the condylar displacement occurs) or imaging findings demonstrating condylar displacement beyond the eminence [5]. This lack of standardised diagnostic criteria undermines efforts to establish clear relationships with TMD.

Beyond diagnostic ambiguity, the possible functional repercussions of TMJ hypermobility remain understudied. Electromyographic evidence suggests that excessive condylar translation may lead to reduced masticatory muscle activity, muscle fatigue and compromised chewing efficiency [6]. Moreover, the medical literature is clear regarding the repercussions of GJH, particularly highlighting chronic pain, fatigue and kinesiophobia [7].

As observed by Braga et al. (2025), the heterogeneity of diagnostic criteria and findings related to TMJ hypermobility suggests that certain diagnostic parameters—whether clinical, anamnestic or imaging‐based—may be more predictive of orofacial functional impairment than others. However, robust multivariate analyses assessing the predictive value of different diagnostic approaches on masticatory function are lacking, as is a comprehensive understanding of the real impact of this condition on the stomatognathic system, particularly regarding its role in the onset and progression of TMD [5].

Identifying reliable predictors of masticatory dysfunction is essential for improving clinical management. Early recognition of individuals at risk could facilitate tailored interventions aimed at preventing progression to pain and functional disability. This is particularly relevant since current diagnostic frameworks, such as the Diagnostic Criteria for Temporomandibular Disorders (DC/TMD), rely heavily on patient self‐report of locking events and do not account for possible subclinical hypermobility [8].

Therefore, the present study aimed to investigate which diagnostic methods for TMJ hypermobility best predict factors related to masticatory dysfunction and the clinical and functional repercussions of TMD. By employing regression analyses, the relative predictive power of clinical (assisted and unassisted maximum opening, lateral condylar jump), anamnestic (history of open‐locking) and imaging parameters was assessed on clinical, functional and psychosocial variables.

## 2 Methods

This research was approved by the Research Ethics Committee of the Bauru School of Dentistry, University of Sao Paulo, Bauru, São Paulo, Brazil (CCAE:64579222.0.0000.5417/No.: 6.296.473). All individuals were informed about the research purposes and signed a voluntary informed consent form. This observational cross‐sectional study was conducted following the Helsinki Declaration and the recommendations of the Strengthening the Reporting of Observational Studies in Epidemiology (Strobe) guidelines [9].

Participants were adults aged between 18 and 40 years evaluated at the Orofacial Pain Clinical Research Center of the Bauru School of Dentistry between September 2023 and July 2025. Mouth opening was assessed following the standardised procedures of the DC/TMD protocol using a conventional millimetre ruler. Three measures were obtained: pain‐free mouth opening (maximum opening without pain or without increasing current pain), unassisted maximum mouth opening (maximum opening regardless of pain) and assisted maximum mouth opening (the examiner applied gentle additional pressure with the fingers on the incisors, stopping immediately if the participant signalled discomfort). In all cases, the measurement corresponded to the distance between the incisal edges of the maxillary and mandibular central incisors, plus the overbite value when present. For analysis purposes, maximum mouth opening was classified using a 55 mm threshold, reflecting the AAOP upper bound (~40–55 mm) for normal opening. As no validated cut‐off exists for TMJ hypermobility, both unassisted and assisted opening were dichotomized as ≤ 55 mm (reduced/normal) versus > 55 mm (wide). Also according to the DC/TMD Axis I framework, TMD can be categorised into pain‐related disorders and intra‐articular disorders [8]. Pain‐related TMDs include conditions such as myalgia, arthralgia and headache attributed to TMD, whereas intra‐articular TMDs comprise disc displacement, degenerative joint disease and subluxation. This distinction has been applied in epidemiological and clinical studies, allowing for separate analyses of pain‐related and joint‐related conditions [10].

In the present study, the ‘painful TMD’ group comprised participants who met only the diagnostic criteria for pain‐related TMD according to the DC/TMD, the ‘dysfunction TMD’ group included those diagnosed exclusively with intra‐articular disorders, and the ‘painful plus dysfunction’ group consisted of individuals meeting diagnostic criteria for both categories simultaneously. The sample (*n* = 126) was equally divided into two subgroups of 63 participants (with a history of open‐locking and those without such history). Open‐locking history was categorized into five frequency levels: never, once in a lifetime, once a year, once a month and more than once a month. This subdivision was made to investigate in a more targeted way the possible impact of the frequency of open locking on TMD outcomes. Bivariate analyses using these categories were prespecified as exploratory/screening procedures to describe distributions and identify candidate predictors; primary inference relied on multivariable binary logistic regression, in which open‐locking history was coded dichotomously (any history vs. none). Within the two subgroups (with and without history of open‐locking), the proportions of the three diagnostic subtypes (painful, dysfunctional and combined) were similar. The categorisation of the participants into three DC/TMD groups was made to capture the full TMD spectrum: purely intra‐articular (e.g., disc displacement with reduction), exclusively pain‐related and combined, representing varying levels of severity and complexity within the sample.

Exclusion criteria included previous TMJ surgery, facial trauma, systemic conditions affecting joint mobility (e.g., heritable connective tissue disorders), current orthodontic treatment, TMJ degenerative joint disease and disc displacement without reduction. During the clinical examination, the presence or absence of lateral condylar jump was assessed from a frontal view while the participant performed maximum mouth opening. Lateral condylar jump is defined as the abrupt jump of the condyle beyond the eminence during terminal opening, a phenomenon also referred to in the literature as jerky movements or abrupt condylar skips, reflecting transient instability typical of TMJ hypermobility [2, 11, 12]. The examiner recorded this finding dichotomously (present/absent).

All clinical examinations were made by a single examiner (SPB) according to the clinical examination that followed the official Brazilian Portuguese version of DC/TMD– Axis I [13, 14]. Participants with orofacial pain were classified according to the International Classification of Orofacial Pain (ICOP 2020) [15].

Painful and non‐painful orofacial symptoms (pain, fatigue, weakness, stiffness and joint instability) were assessed using a visual analog scale (VAS) adapted from previously described methods for chronic pain assessment in the DC/TMD protocol. Participants rated current intensity, average intensity over the past 30 days and highest intensity in the past 30 days; then, the final score was the mean of these three ratings [14]. Before the assessment, the definition of each symptom was read aloud to the participants to ensure a clear and consistent understanding.

Pain was defined as an unpleasant sensory and emotional experience associated with, or resembling that associated with, actual or potential tissue damage [16]. Muscle fatigue was defined as the failure to maintain the necessary force during repeated or sustained contractions. In the face, it can manifest as a progressive reduction in strength during prolonged activities, such as chewing firm food or speaking continuously [17]. Weakness was defined as a reduced strength in one or more muscles [18]. Stiffness was defined as resistance provided by tissue, joint or limb to a change in shape and position [19]. Joint instability was defined as the patient's self‐reported perception that the joint feels loose, unstable or prone to giving way, slipping or locking, regardless of objective evidence of laxity. This experience is often described as a feeling of looseness, dislocation or lack of confidence in the joint and represents a distinct clinical dimension not fully captured by objective tests. This phenomenon has been consistently reported across different conditions, such as knee osteoarthritis and anterior cruciate ligament injury, where patients describe functional insecurity that cannot always be explained by objective findings [20, 21]. Participants were instructed to extrapolate the concepts previously read aloud to the orofacial region.

All participants were assessed for the presence of GJH using a series of simple tests to evaluate joint range of motion, known as the Beighton score, which is a modified version of a technique originally developed by Carter and Wilkinson [22, 23]. A recent systematic review concludes that, in adults, the Beighton score with a cutoff point of 5/9 is the recommended approach for clinical use [24].

The mechanical quantitative sensory test for pressure pain threshold (PPT) represents the point at which increasing pressure applied to a specific area becomes unpleasant or painful for the individual. PPT was assessed using algometry, performed with a computerised pressure algometer, the Medoc AlgoMed (Medoc Ltd. Advanced Medical Systems, Israel), which provides real‐time visual and auditory feedback on the computer screen. Three sequential measurements were taken at each site, and the final PPT value was calculated as the mean of these three measurements [25, 26]. Assessments were conducted bilaterally over the TMJ area and on the masseter or anterior temporalis muscles.

Masticatory muscle fatigue was objectively assessed using the digital bite‐force dynamometer (Kratos, Cotia, São Paulo, Brazil). Initially, participants performed three maximal voluntary clenching (MVC) trials, each lasting 4 s, with 5 min rest intervals between trials. The highest value obtained was recorded as the pre‐fatigue maximum bite force (MBF). After a 10 min rest period, participants completed a fatigue test consisting of sustained unilateral clenching at 30% of their pre‐fatigue MBF. This task was guided by real‐time visual feedback displayed on a computer screen and verbal encouragement to help maintain the target force. The test was finished when the bite force dropped by 10% or more below the target for more than three consecutive seconds. The total time sustained at the target force (endurance time) was recorded. Immediately after the fatigue task, a post‐fatigue MBF was performed and the percentage change in MBF was calculated using the formula: (pre‐MBF−post‐MBF)/pre‐MBF × 100 [27].

Using a portable ultrasound device (Philips Ultrasound. Lumify Diagnostic Ultrasound System. Bothell, WA: Philips Ultrasound; 2023), the thickness of the TMJ capsule was measured bilaterally to investigate findings indicative of painful and/or inflammatory TMJ conditions. Additionally, the thickness of the masseter muscle was assessed during both contraction and relaxation. Each final value was calculated as the mean of three repeated measurements [28, 29, 30].

**FIGURE 1:**
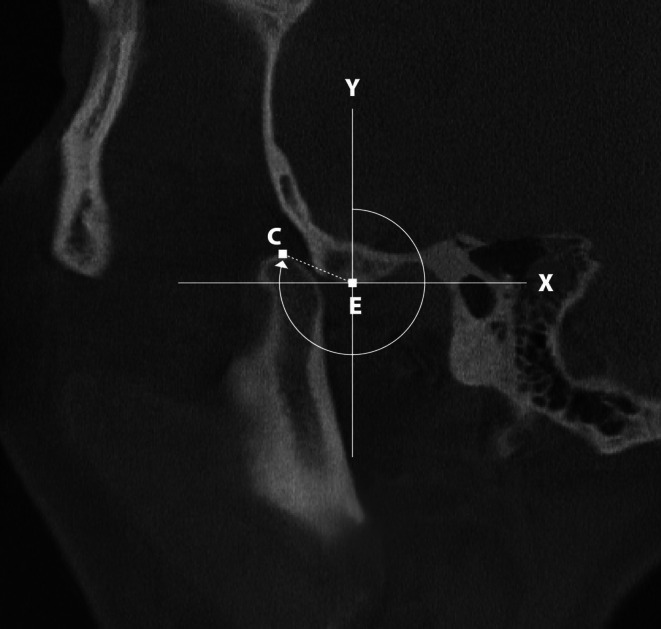
Measurement of the condylar angle (°) according to the method of Kalaykova et al. (2006), adapted for CBCT images. In the parasagittal slice, point E corresponds to the most inferior aspect of the articular eminence crest, and point C corresponds to the most superior aspect of the condylar cortex. A vertical line (*Y*‐axis) and its perpendicular horizontal line (*X*‐axis) were drawn from point E to establish a Cartesian coordinate system. The condylar angle was defined as the angle between the EC line and the vertical axis.

Cone‐beam computed tomography (CBCT) scans with participants in maximum mouth opening position were performed to quantify the condylar position relative to the articular eminence. The condylar angle (°) (Figure 1) was measured following the method described by Kalaykova et al. (2006), adapted for CBCT images. In the parasagittal slice that best displayed the condyle–articular eminence relationship, two anatomical landmarks were identified: E (eminence point): the most inferior point of the articular eminence crest; C (condylar point): the most superior point of the condylar cortex. From point E, a vertical line (*Y*‐axis) and a horizontal line (*X*‐axis) perpendicular to it were constructed, establishing a Cartesian coordinate system. The condylar angle was defined as the angle between the line connecting points E and C (EC) and the vertical *Y*‐axis. Larger angles indicate more anterior and/or superior condylar positioning relative to the eminence crest, with values exceeding 180° representing positions anterior to the crest [31]. Given the absence of an established threshold, the condylar angle was dichotomized at the sample median for modeling (lower vs. higher angle), as detailed in the following data evaluation and statistical analyses section.

The translated version of the Jaw Functional Limitation Scale (JFLS) from the DC/TMD was used to assess the degree of mandibular functional limitation in the participants [32]. The level of pain catastrophizing was assessed using the Pain Catastrophizing Scale (PCS) [33] and the phenomenon of hypervigilance was evaluated using the Pain Vigilance and Awareness Questionnaire (PVAQ) [34]. Finally, the level of mandibular kinesiophobia was assessed using the Tampa Scale of Kinesiophobia for Temporomandibular Disorders (TSK‐TMD) [35].

## 3 Data Evaluation and Statistical Analyses

The predictors evaluated included anamnestic (history of open‐locking), imaging (right and left condylar angle) and clinical measures (maximum unassisted and assisted mouth opening, lateral condylar jump). All other variables were considered as dependent outcomes. Data were expressed as mean ± standard deviation, with normality assessed using the Kolmogorov–Smirnov test. Comparisons were performed with the Kruskal–Wallis/Dunn or Mann–Whitney tests. After categorisation at the median, categorized and categorical variables were presented as absolute and relative frequencies and analyzed for associations using Fisher's exact test or Pearson's chi‐square test. Variables with *p* < 0.200 in bivariate analyses were entered into a multiple binary logistic regression model. All analyses were conducted at a 95% confidence level using SPSS software, version 20.0 for Windows.

## 4 Results

### 4.1 Bivariate Regression

In the bivariate analyses, associations were examined according to four main variables of interest: the frequency of open‐locking episodes, right and left condylar angles, unassisted and assisted maximum mouth opening and the presence of lateral condylar jump.

In the first bivariate analysis (*p < 0.05, Fisher's exact test or Pearson's chi‐square test (*n*, %); *p* < 0.05, Kruskal–Wallis test with Dunn's post hoc (mean ± SD)), frequent open‐locking (≥ 1/month) was associated with lower pain‐free mouth opening (≤40 mm; *p* = 0.010) but greater assisted opening (> 55 mm; *p* = 0.024), indicating preserved passive mobility despite pain‐related restriction. It was also linked to the presence of lateral condylar jump (*p* = 0.005), DC/TMD TMJ subluxation diagnosis (*p* < 0.001), painful muscular TMD (*p* = 0.016) and ICOP‐defined TMJ pain attributed to subluxation (*p* < 0.001). Higher orofacial symptom scores—pain (*p* = 0.024), stiffness (*p* = 0.018), and instability (*p* < 0.001)—as well as greater mandibular kinesiophobia (*p* < 0.001) and increased functional limitation (*p* = 0.006), particularly in wide‐opening tasks (*p* < 0.001), were also observed in participants with frequent episodes of open‐locking (Table S1).

In the second bivariate analysis (*p < 0.05, Fisher's exact test or Pearson's chi‐square test (*n*, %); *p < 0.05, Mann–Whitney test (mean ± SD)), the condylar angles > 280° (right and left) were associated with greater pain‐free, unassisted and assisted maximum mouth opening (all *p* < 0.001), higher frequency of lateral condylar jump (*p* = 0.026 right; *p* < 0.001 left), midline deviation (*p* ≤ 0.007) and terminal click (*p* = 0.015 right; *p* = 0.007 left). Higher condylar angles correlated with lower orofacial pain scores (*p*≈0.038–0.039). Left condylar angles > 280° were linked to greater pre‐fatigue bite force (*p* = 0.001). Right condylar angles > 280° were linked to lower hypervigilance (*p* = 0.004). Conversely, right condylar angles ≤ 280° were associated with higher total JFLS scores (*p* = 0.035), indicating greater functional impairment (Table S2).

The third bivariate analysis (*p < 0.05, Fisher's exact test or Pearson's chi‐square test (*n*, %); *p < 0.05, Mann–Whitney test (mean ± SD)) showed greater unassisted and assisted mouth opening (> 55 mm) was associated with larger condylar angles (*p* ≤ 0.038), greater pain‐free opening (*p* < 0.001) and higher PPT values in the TMJ, masseter and temporalis (*p* < 0.05), indicating lower mechanical pain sensitivity. Male sex was related to greater unassisted opening (*p* = 0.040), while smaller opening (≤ 55 mm) was linked to higher orofacial pain scores (*p* = 0.005), greater functional limitation (*p* < 0.001), limitation in wide‐opening tasks (*p* = 0.028) and lower prevalence of lateral condylar jump (*p* ≤ 0.011) and midline deviation (*p* ≤ 0.015). Higher opening values were further associated with greater pre‐ and post‐fatigue MBF (*p* ≤ 0.041) and a larger post‐fatigue decrease (*p* ≤ 0.040), suggesting distinct functional performance profiles (Table S3).

The fourth bivariate analysis (*p < 0.05, Fisher's exact test or Pearson's chi‐square test (*n*, %); *p < 0.05, Mann–Whitney test (mean ± SD)) showed that lateral condylar jump was associated with larger condylar angles—particularly on the left (*p* = 0.011)—and a higher prevalence of condylar angles > 280° (*p* = 0.001). It was linked to more frequent open‐locking episodes (*p* = 0.005), greater unassisted and assisted mouth opening (*p* = 0.024 and *p* < 0.001), midline deviation (*p* < 0.001) and terminal clicking (*p* = 0.033). This sign was also associated with subluxation diagnosis (*p* = 0.005), TMJ pain attributed to subluxation (*p* = 0.040), DDWR with intermittent locking (*p* = 0.027), higher joint instability scores (*p* = 0.003) and greater functional limitation in wide‐opening tasks (*p* = 0.009) (Table S4).

## 5 Multivariate Regression

Multiple binary logistic regression (Table 1) showed that open‐locking episodes predicted subluxation diagnosis (*p* = 0.006; aOR = 16.12) and higher orofacial joint instability scores (*p* = 0.035; aOR = 68.80). Increased condylar angles were associated with greater assisted opening (*p* = 0.007; aOR = 27.79) and midline deviation (*p* = 0.028; aOR = 11.73), while magnification was inversely related (*p* = 0.002; aOR = 0.02). Unassisted maximum mouth opening predicted larger right condylar angle (*p* = 0.005; aOR = 8.76), greater pain‐free opening (*p* < 0.001; aOR = 33.15) and lateral condylar jump (*p* = 0.004; aOR = 12.50). Assisted maximum mouth opening was also associated with pain‐free maximum mouth opening (*p* = 0.004; aOR = 6.37) and lateral condylar jump (*p* = 0.012; aOR = 6.38). Lateral condylar jump predicted functional limitation in wide‐opening tasks (*p* = 0.048; aOR = 5.87).

**TABLE 1:** * Multiple binary logistic regression (*p* < 0.20) assessing the predictive value of open‐locking history, maximum mouth opening (assisted and unassisted), condylar angle, and lateral condylar jump for clinical, diagnostic and self‐reported outcomes.

	*p*	aOR	95% CI	
Open‐locking episodes				
Age	0.097	—	—	—
Pain‐free maximum mouth opening	0.081	—	—	—
Assisted maximum mouth opening	0.184	—	—	—
Lateral condylar jump	1.000	—	—	—
Subluxation diagnosis (DC/TMD)	*0.006*	*16.12*	*3.32*	*78.26*
Muscular TMD diagnosis (DC/TMD)	1.000	—	—	—
Headache attributed to TMD	0.401	—	—	—
Arthralgia	1.000	—	—	—
TMJ pain attributed to subluxation (ICOP)	1.000	—	—	—
Muscle pain diagnosis (ICOP)	1.000	—	—	—
TMJ pain diagnosis (ICOP)	1.000	—	—	—
Orofacial pain (VAS)	0.887	—	—	—
Orofacial stiffness (VAS)	0.245	—	—	—
Orofacial joint instability (VAS)	*0.035*	*68.80*	*1.35*	*3516.72*
Rumination	0.072	—	—	—
Total catastrophizing score	0.053	—	—	—
Mandibular kinesiophobia	0.121	—	—	—
JFLS score	0.844	—	—	—
JFLS items 7 and 12	0.534	—	—	—
Increased condylar angles				
Pain‐free maximum mouth opening	0.175	—	—	—
Unassisted maximum mouth opening	0.085	—	—	—
Assisted maximum mouth opening	*0.007*	*27.79*	*2.48*	*310.98*
Lateral condylar jump	0.074	—	—	—
Midline deviation during opening	*0.028*	*11.73*	*1.31*	*105.32*
Terminal click	0.106	—	—	—
Subluxation diagnosis (DC/TMD)	1.000	—	—	—
Arthralgia	0.589	—	—	—
TMJ pain diagnosis (ICOP)	0.522	—	—	—
DDWR with intermittent locking (DC/TMD)	0.299	—	—	—
Orofacial pain (VAS)	0.872	—	—	—
Right TMJ PPT (kgf)	0.627	—	—	—
Left TMJ PPT (kgf)	0.797	—	—	—
Right masseter PPT (kgf)	0.084	—	—	—
Left masseter PPT (kgf)	0.363	—	—	—
Pre‐fatigue MBF	0.449	—	—	—
Left TMJ articular capsule	0.947	—	—	—
Right TMJ articular capsule	0.070	—	—	—
Right masseter (contraction)	0.833	—	—	—
Left masseter (contraction)	0.189	—	—	—
Helplessness	0.332	—	—	—
Magnification	*0.002*	*0.02*	*0.01*	*0.20*
Total catastrophizing score	0.785	—	—	—
Mandibular kinesiophobia	0.966	—	—	—
Hypervigilance score	0.079	—	—	—
JFLS score	0.621	—	—	—
JFLS items 7 and 12	0.098	—	—	—
Unassisted maximum mouth opening				
Sex	0.657	—	—	—
Right condylar angle	*0.005*	*8.76*	*1.93*	*39.64*
Left condylar angle	0.063	—	—	—
Open‐locking episodes	0.650	—	—	—
Pain‐free maximum mouth opening	*< 0.001*	*33.15*	*6.03*	*182.11*
Unassisted maximum mouth opening	1.000	—	—	—
Assisted maximum mouth opening	1.000	—	—	—
Lateral condylar jump	*0.004*	*12.50*	*2.25*	*69.53*
Midline deviation during opening	0.849	—	—	—
Terminal click	0.634	—	—	—
Subluxation diagnosis (DC/TMD)	0.365	—	—	—
Muscular TMD diagnosis (DC/TMD)	1.000	—	—	—
TMJ pain attributed to subluxation (ICOP)	1.000	—	—	—
Muscle pain diagnosis (ICOP)	1.000	—	—	—
DDWR with intermittent locking (DC/TMD)	1.000	—	—	—
Orofacial pain (VAS)	0.403	—	—	—
Orofacial fatigue (VAS)	0.267	—	—	—
Orofacial joint instability (VAS)	0.223	—	—	—
Right TMJ PPT (kgf)	1.000	—	—	—
Right TMJ PPT (kgf)	1.000	—	—	—
Right masseter PPT (kgf)	1.000	—	—	—
Left masseter PPT (kgf)	1.000	—	—	—
Right temporalis PPT (kgf)	1.000	—	—	—
Left temporalis PPT (kgf)	1.000	—	—	—
Pre‐fatigue MBF	0.515	—	—	—
Endurance time	0.062	—	—	—
Post‐ fatigue MBF	0.224	—	—	—
Percentage change in MBF	0.257	—	—	—
Left TMJ articular capsule	0.925	—	—	—
Right TMJ articular capsule	0.096	—	—	—
Right masseter (contraction)	0.378	—	—	—
Left masseter (contraction)	0.130	—	—	—
Magnification	0.718	—	—	—
Total catastrophizing score	0.325	—	—	—
JFLS score	0.637	—	—	—
JFLS items 7 and 12	0.195	—	—	—
Assisted maximum mouth opening				
Sex	0.746	—	—	—
Right condylar angle	0.227	—	—	—
Left condylar angle	0.664	—	—	—
Open‐locking episodes	0.553	—	—	—
Pain‐free maximum mouth opening	*0.004*	*6.37*	*1.81*	*22.41*
Unassisted maximum mouth opening	1.000	—	—	—
Assisted maximum mouth opening	1.000	—	—	—
Lateral condylar jump	*0.012*	*6.38*	*1.51*	*26.94*
Midline deviation during opening	0.707	—	—	—
Terminal click	0.493	—	—	—
Subluxation diagnosis (DC/TMD)	0.571	—	—	—
Muscular TMD diagnosis (DC/TMD)	1.000	—	—	—
TMJ pain attributed to subluxation (ICOP)	1.000	—	—	—
Muscle pain diagnosis (ICOP)	1.000	—	—	—
DDWR with intermittent locking (DC/TMD)	1.000	—	—	—
Orofacial pain (VAS)	0.371	—	—	—
Orofacial fatigue (VAS)	0.885	—	—	—
Orofacial joint instability (VAS)	0.963	—	—	—
Right TMJ PPT (kgf)	1.000	—	—	—
Left TMJ PPT (kgf)	1.000	—	—	—
Right masseter PPT (kgf)	1.000	—	—	—
Left masseter PPT (kgf)	1.000	—	—	—
Right temporalis PPT (kgf)	1.000	—	—	—
Left temporalis PPT (kgf)	1.000	—	—	—
Pre‐fatigue MBF	0.715	—	—	—
Endurance time	0.280	—	—	—
Post‐ fatigue MBF	0.838	—	—	—
Percentage change in MBF	0.768	—	—	—
Left TMJ articular capsule	0.437	—	—	—
Right TMJ articular capsule	0.422	—	—	—
Right masseter (contraction)	0.783	—	—	—
Left masseter (contraction)	0.650	—	—	—
Magnification	0.747	—	—	—
Total catastrophizing score	0.879	—	—	—
JFLS score	0.905	—	—	—
JFLS items 7 and 12	0.199	—	—	—
Lateral condylar jump				
Right condylar angle	0.726	—	—	—
Left condylar angle	0.110	—	—	—
Open‐locking episodes	1.000	—	—	—
Unassisted maximum mouth opening	0.318	—	—	—
Assisted maximum mouth opening	0.723	—	—	—
Midline deviation during opening	0.093	—	—	—
Terminal click	1.000	—	—	—
Subluxation diagnosis (DC/TMD)	1.000	—	—	—
Muscular TMD diagnosis (DC/TMD)	1.000	—	—	—
Arthralgia	0.122	—	—	—
TMJ pain attributed to subluxation (ICOP)	1.000	—	—	—
TMJ pain diagnosis (ICOP)	1.000	—	—	—
Disc displacement with reduction (DDWR) (DC/TMD)	0.144	—	—	—
DDWR with intermittent locking (DC/TMD)	0.261	—	—	—
Orofacial Stiffness (VAS)	0.359	—	—	—
Orofacial joint instability (VAS)	0.868	—	—	—
Right TMJ PPT (kgf)	0.840	—	—	—
Left TMJ PPT (kgf)	0.646	—	—	—
Right masseter PPT (kgf)	0.078	—	—	—
Left masseter PPT (kgf)	0.209	—	—	—
Subjective fatigue (VAS) (post‐fatigue)	0.103	—	—	—
Rumination	0.605	—	—	—
Total catastrophizing score	0.738	—	—	—
JFLS items 7 and 12	*0.048*	*5.87*	*1.01*	*33.99*

Abbreviations: 95% CI, 95% confidence interval of the ORa; ORa, adjusted odds ratio.

* *p* < 0.05, multinomial logistic regression. Italic *p*‐values indicate statistical significance at *p* < 0.05.

## 6 Discussion

These findings should be interpreted within the broader context of joint laxity and hypermobility disorders since excessive joint laxity is frequent in young individuals and has been linked to various musculoskeletal injuries. In symptomatic cases, it may present with chronic pain, fatigue, kinesiophobia and joint instability, reflecting its multisystemic nature [7, 36]. Evidence on the effects of GJH and TMJ hypermobility on the stomatognathic system is inconsistent, with studies reporting both positive and absent associations with TMD, pain, open‐locking and functional limitations. These discrepancies likely arise from the lack of standardized diagnostic criteria and heterogeneous assessment methods, hindering a clear understanding of the functional impact of condylar hypermobility [5].

In the first bivariate analysis, frequent open‐locking (≥ 1/month) was associated with reduced pain‐free mouth opening (≤ 40 mm), consistent with Dinsdale et al. (2022) [37] who reported decreased active mouth opening in intra‐articular TMD. In contrast, assisted opening was greater in these patients, suggesting preserved passive mobility consistent with TMJ hypermobility [4]. This finding highlights the potential functional impact of open‐locking on pain, while indicating that assisted mandibular mobility remains preserved. The lateral condylar jump was more frequent in recurrent open‐locking, aligning with its potential as a clinical sign of TMJ hypermobility, though this association did not remain in the multivariate model.

Open‐locking was also linked to DC/TMD TMJ subluxation, TMJ pain attributed to subluxation (according to the ICOP classification), painful muscle TMD and higher pain, stiffness and joint instability scores. Higher mandibular kinesiophobia and greater jaw function limitation, particularly in wide‐opening tasks, were also observed. Dinsdale et al. (2024) emphasized that, in intra‐articular TMD, perceived functional limitations are more closely related to stiffness and kinesiophobia than to objective deficits in mobility, suggesting altered motor control or proprioception as possible mechanisms [37]. Manfredini (2007), in turn, described the stabilizing role of the masticatory muscles, although the notion of a compensatory muscular response remains controversial [38]. In this context, the association of open‐locking with subluxation, painful muscular TMD and higher pain, stiffness and instability scores suggests that both articular mechanics and adaptive muscular responses may contribute to the clinical impact of joint instability.

These findings prompt reflection, as Okeson and Bell describe TMJ subluxation as a non‐pathological anatomical variation, whereas the DC/TMD framework classifies it as a distinct TMD subtype [2, 8]. This discrepancy underscores the ongoing debate over whether subluxation should be regarded as a benign anatomical feature or a dysfunction warranting clinical management.

The multivariate analysis confirmed that a history of open‐locking is a strong predictor of DC/TMD‐defined TMJ subluxation, reinforcing its role as a hallmark of TMJ hypermobility. Since both TMJ subluxation and luxation represent potential clinical consequences of excessive condylar translation, the occurrence of open‐locking often constitutes the critical event that prompts patients to seek treatment. However, the DC/TMD framework restricts the diagnostic classification of subluxation to episodes occurring within the last 30 days which may underestimate the clinical relevance of individuals who experience open‐locking infrequently or who report only remote episodes [8]. This narrow temporal criterion risks overlooking patients with clear biomechanical vulnerability and a history consistent with joint instability. From this perspective, open‐locking history, independent of temporality, should be considered a meaningful clinical indicator of underlying TMJ hypermobility, with potential implications for identifying patients with increased susceptibility to TMJ instability, guiding preventive counselling and tailoring management strategies aimed at minimising the risk of progression to recurrent TMJ subluxation or dislocation.

Furthermore, in both bivariate and multivariate analyses, a history of open‐locking was associated with increased self‐reported orofacial joint instability, highlighting the clinical relevance of non‐painful symptoms. Such instability likely reflects deficient passive stabilising mechanisms due to ligamentous laxity and may precede TMD pain [7]. Baad‐Hansen et al. (2019) noted that non‐painful musculoskeletal signs remain underexplored despite potential predictive value [39]. In this sample the functional impact of open‐locking appeared limited to its association with TMJ subluxation and joint instability, suggesting the need to distinguish mechanical manifestations of TMJ hypermobility from pain‐related and functional impairments arising from other mechanisms of TMD.

These findings question whether TMJ hypermobility, represented by open‐locking, has a direct role in TMD pathophysiology. In this sample, open‐locking history was associated only with TMJ subluxation and showed no link to GJH, contrasting with studies reporting broader associations with articular TMD [40, 41, 42]. However, such discrepancies may also reflect differences in diagnostic criteria, sample characteristics and study design.

In the second bivariate analysis, greater condylar angles (> 280°) were associated with greater pain‐free, unassisted and assisted mouth opening, as well as higher frequencies of lateral condylar jump, midline deviation and terminal click, a clinical sign of TMJ hypermobility. Although studies in asymptomatic individuals have not demonstrated a consistent relationship between eminence morphology and mouth opening, evidence from hypermobile patients indicates that certain anatomical features may predict excessive opening, suggesting that the role of anatomy becomes more apparent in this context [43, 44]. Furthermore, higher condylar angles were linked to lower orofacial pain scores indicating no increase in facial pain sensitivity. Left condylar angles > 280° were also associated with greater pre‐fatigue bite force, and right condylar angles > 280° with lower hypervigilance, while right condylar angles ≤ 280° correlated with greater functional limitation, suggesting a limited and possibly protective effect on these factors. Our findings are consistent with the broader literature indicating that TMD pain arises from multifactorial biopsychosocial processes, and that anatomical features alone, such as TMJ morphology, often show weak correlation with pain severity [45].

While bivariate analysis linked increased condylar angles to multiple functional, clinical and psychosocial variables, multivariate regression retained only assisted mouth opening, midline deviation and lower pain magnification as independent associations. These findings suggest that larger condylar angles primarily predict functional mobility rather than pain sensitivity or psychosocial impairment, possibly reflecting an anatomical variation that facilitates mandibular motion without increasing dysfunction risk. Although some classifications cite anterior condylar displacement as a complementary sign of TMJ subluxation or hypermobility [46, 47, 48], the present findings are consistent with the study of Kalaykova (2006) since they do not support condylar angle or condylar displacement as a predictor of TMJ dysfunction [31].

In the third bivariate analysis, greater maximum unassisted and assisted mouth opening (> 55 mm) was associated with a larger condylar angle, higher pain‐free opening, higher PPT in TMJ and masticatory muscles, more frequent hypermobility signs (lateral condylar jump, midline deviation), lower pain scores and greater pre‐ and post‐fatigue bite force, though with a larger percentage loss after fatigue. This functional profile aligns with a previous study that showed masticatory muscle activity decreases with increasing severity of TMJ hypermobility, suggesting that excessive opening may alter muscle performance and endurance [6]. Conversely, reduced mouth opening (≤ 55 mm) was linked to smaller condylar angles, more pain, greater functional limitation and fewer hypermobility signs. In multivariate analysis, however, unassisted mouth opening remained associated only with greater right condylar angle, pain‐free opening and lateral condylar jump, while assisted mouth opening remained associated only with pain‐free opening and lateral condylar jump—indicating that higher maximum opening values mainly predict mobility‐related parameters rather than pain sensitivity or broader dysfunction.

In the fourth bivariate analysis, lateral condylar jump was associated with larger condylar angles (> 280°), greater unassisted and assisted opening, more frequent open‐locking, midline deviation, terminal clicking, subluxation diagnosis (DC/TMD), TMJ pain attributed to subluxation (ICOP), higher joint instability scores and greater functional limitation in wide‐opening tasks. Taken together, these associations suggest that TMJ hypermobility can reflect both anatomical predispositions and functional adaptations, consistent with mechanistic explanations proposed in previous studies [6, 43].

The clinical sign lateral condylar jump describes the abrupt jump of the condyle beyond the eminence during terminal opening, a phenomenon also referred to in the literature as jerky movements or abrupt condylar skips, reflecting transient instability typical of TMJ hypermobility [2, 11]. In the multivariate analysis, lateral condylar jump remained independently associated only with a higher limitation in tasks requiring extreme mouth opening. This finding is consistent with previous evidence highlighting its diagnostic value for TMJ hypermobility. Tuijt et al. [12] suggest that instability occurs when muscular compensation is required to maintain condylar stability at extreme positions, highlighting the need for diagnostic approaches that integrate clinical, imaging and subjective perception of instability. Such results indicate that lateral condylar jump may serve as a specific marker of mechanical TMJ instability rather than a broad predictor of dysfunction, reinforcing its clinical utility while suggesting that its impact depends on the analytical framework applied. A possible explanation is the multivariate model simultaneously accounted for anamnestic, anatomical and functional predictors, thereby reducing the independent contribution of this clinical sign. While lateral condylar jump reflects mechanical instability, its role as a predictor of broader dysfunction may be context‐dependent and less robust when other mobility‐related parameters are considered.

## 7 Conclusions

Open‐locking history emerged as a strong marker of both TMJ subluxation diagnosis and subjective orofacial joint instability. Larger condylar angles were associated with greater mandibular mobility, midline deviation and lower pain catastrophizing. Increased maximum mouth opening, whether assisted or unassisted, predicted greater pain‐free mobility but also a higher likelihood of lateral condylar jump, which in turn was a predictor of functional limitation in tasks requiring wide mandibular opening.

It can be inferred that TMJ hypermobility, when assessed through clinical, functional and imaging indicators, does not appear to exert a substantial impact on the overall presentation of TMD or on their broader clinical and functional outcomes. Furthermore, indicators of TMJ hypermobility—clinical, functional and imaging‐based—capture distinct aspects of the phenomenon, and no single marker is capable of predicting the full spectrum of manifestations.

## Author Contributions

S.P.B. conceived and designed the study, collected the data, performed the analyses and drafted the manuscript. D.M.F., M.E.S.B., A.C.C.F.C., A.M. and P.C.R.C. contributed to the critical revision of the manuscript. All authors approved the final version.

## Funding

This work was supported by Coordenação de Aperfeiçoamento de Pessoal de Nível Superior.

## Conflicts of Interest

The authors declare no conflicts of interest.

## Supporting information


**Table S1:** Bivariate associations between the frequency of open‐locking episodes and clinical, functional and psychosocial variables. *p* < 0.05, Fisher's exact test or Pearson's chi‐square test (*n*, %); *p* < 0.05, Kruskal–Wallis test with Dunn's post hoc (mean ± SD).

**Table S2:** Bivariate associations between the right and left TMJ angle and clinical, functional and psychosocial variables. *p < 0.05, Fisher's exact test or Pearson's chi‐square test (*n*, %); *p < 0.05, Mann–Whitney test (mean ± SD).

**Table S3:** Bivariate analysis considering maximum assisted and unassisted mouth opening as predictors of clinical, functional and psychosocial outcomes. *p < 0.05, Fisher's exact test or Pearson's chi‐square test (*n*, %); *p < 0.05, Mann–Whitney test (mean ± SD).

**Table S4:** Bivariate analysis considering the presence of lateral condylar jump as a predictor of clinical, functional and psychosocial variables. *p < 0.05, Fisher's exact test or Pearson's chi‐square test (*n*, %); *p < 0.05, Mann–Whitney test (mean ± SD).

## Data Availability

The datasets generated and/or analysed during the current study are available from the corresponding author on reasonable request.
