# Supplementary material for: Predictive Value of Diagnostic Methods for TMJ Hypermobility in the Associated Clinical and Functional Features of Temporomandibular Disorders: A Regression Study
Source: J Oral Rehabil. 2025 Nov 27;53(3):673–84. doi: 10.1111/joor.70120 (PMC12902195; doi:10.1111/joor.70120)
Supplement: Supplementary file 1 — Table S1: Bivariate associations between the frequency of open‐locking episodes and clinical, functional and psychosocial variables. p < 0.05, Fisher's exact test or Pearson's chi‐square test (n, %); p < 0.05, Kruskal–Wallis test with Dunn's post hoc (mean ± SD). [file JOOR-53-673-s001.docx]

|  | **Open-locking episodes** | | | | |  |
| --- | --- | --- | --- | --- | --- | --- |
|  | **Never** | **Once in a lifetime** | **Once a year** | **Once a month** | **More than once a month** | **p-value** |
| **Sex** |  |  |  |  |  |  |
| Male | 13 (21.3%) | 5 (17.9%) | 3 (33.3%) | 5 (35.7%) | 6 (42.9%) | 0,318 |
| Female | 48 (78.7%) | 23 (82.1%) | 6 (66.7%) | 9 (64.3%) | 8 (57.1%) |  |
| **Age** | 27.25±5.66 | 28.61±5.91 | 28.11±5.80 | 24.36±5.27 | 25.93±5.27 | 0,190 |
| Up to 25 | 29 (47.5%) | 8 (28.6%) | 4 (44.4%) | 9 (64.3%) | 8 (57.1%) | 0,194 |
| Over 25 | 32 (52.5%) | 20 (71.4%) | 5 (55.6%) | 5 (35.7%) | 6 (42.9%) |  |
| **Right TMJ angle** | 274.26±25.76 | 271.11±40.65 | 272.85±15.48 | 269.62±45.29 | 287.11±9.63 | 0,547 |
| Up to 280º | 27 (44.3%) | 10 (35.7%) | 5 (55.6%) | 6 (42.9%) | 3 (21.4%) | 0,460 |
| Over 280º | 34 (55.7%) | 18 (64.3%) | 4 (44.4%) | 8 (57.1%) | 11 (78.6%) |  |
| **Left TMJ angle** | 273.38±26.40 | 274.41±35.82 | 274.08±19.62 | 268.13±49.82 | 284.34±10.82 | 0,712 |
| Up to 280º | 28 (45.9%) | 12 (42.9%) | 4 (44.4%) | 5 (35.7%) | 6 (42.9%) | 0,974 |
| Over 280º | 33 (54.1%) | 16 (57.1%) | 5 (55.6%) | 9 (64.3%) | 8 (57.1%) |  |
| **Pain-free maximum mouth opening** | 43.61±12.23 | 43.11±10.59 | 43.00±10.48 | 35.93±12.92 | 39.57±14.30 | 0,252 |
| Up to 40 | 17 (27.9%) | 13 (46.4%) | 3 (33.3%) | 9 (64.3%)* | 10 (71.4%)* | ***0,010*** |
| Over 40 | 44 (72.1%)* | 15 (53.6%)* | 6 (66.7%)* | 5 (35.7%) | 4 (28.6%) |  |
| **Unassisted maximum mouth opening** | 54.48±7.12 | 54.79±7.62 | 59.11±6.55 | 53.14±6.49 | 57.29±7.75 | 0,237 |
| Up to 55 | 34 (55.7%) | 14 (50.0%) | 3 (33.3%) | 9 (64.3%) | 7 (50.0%) | 0,654 |
| Over 55 | 27 (44.3%) | 14 (50.0%) | 6 (66.7%) | 5 (35.7%) | 7 (50.0%) |  |
| **Assisted maximum mouth opening** | 57.02±6.68 | 58.07±6.31 | 61.67±6.65 | 58.86±4.24 | 61.71±7.26 | 0,069 |
| Up to 55 | 27 (44.3%)* | 11 (39.3%)* | 0 (0.0%) | 2 (14.3%) | 3 (21.4%)* | ***0,024*** |
| Over 55 | 34 (55.7%) | 17 (60.7%) | 9 (100.0%)* | 12 (85.7%)* | 11 (78.6%) |  |
| **Lateral condylar jump** |  |  |  |  |  |  |
| No | 20 (32.8%)* | 3 (10.7%)* | 2 (22.2%)* | 0 (0.0%) | 0 (0.0%) | ***0,005*** |
| Yes | 41 (67.2%) | 25 (89.3%) | 7 (77.8%) | 14 (100.0%)* | 14 (100.0%)* |  |
| **Midline deviation during opening** |  |  |  |  |  |  |
| No | 12 (19.7%) | 5 (17.9%) | 1 (11.1%) | 1 (7.1%) | 0 (0.0%) | 0,347 |
| Yes | 49 (80.3%) | 23 (82.1%) | 8 (88.9%) | 13 (92.9%) | 14 (100.0%) |  |
| **Terminal click** |  |  |  |  |  |  |
| No | 56 (91.8%) | 22 (78.6%) | 8 (88.9%) | 12 (85.7%) | 12 (85.7%) | 0,537 |
| Yes | 5 (8.2%) | 6 (21.4%) | 1 (11.1%) | 2 (14.3%) | 2 (14.3%) |  |
| **Subluxation diagnosis (DC/TMD)** |  |  |  |  |  |  |
| No | 61 (100.0%)* | 28 (100.0%)* | 8 (88.9%)* | 3 (21.4%) | 1 (7.1%) | ***<0,001*** |
| Yes | 0 (0.0%) | 0 (0.0%) | 1 (11.1%) | 11 (78.6%)* | 13 (92.9%)* |  |
| **Muscular TMD diagnosis (DC/TMD)** |  |  |  |  |  |  |
| None | 20 (32.8%)* | 5 (17.9%)* | 2 (22.2%)* | 5 (35.7%)* | 1 (7.1%) | ***0,016*** |
| Local myalgia | 19 (31.1%) | 3 (10.7%) | 2 (22.2%) | 1 (7.1%) | 3 (21.4%)* |  |
| Myofascial pain | 12 (19.7%) | 13 (46.4%) | 4 (44.4%) | 1 (7.1%) | 5 (35.7%)* |  |
| Myofascial pain with referral | 10 (16.4%) | 7 (25.0%) | 1 (11.1%) | 7 (50.0%) | 5 (35.7%)* |  |
| **Headache attributed to TMD** |  |  |  |  |  |  |
| No | 40 (65.6%) | 12 (42.9%) | 3 (33.3%) | 7 (50.0%) | 6 (42.9%) | 0,135 |
| Yes | 21 (34.4%) | 16 (57.1%) | 6 (66.7%) | 7 (50.0%) | 8 (57.1%) |  |
| **Arthralgia** |  |  |  |  |  |  |
| No | 36 (59.0%) | 9 (32.1%) | 4 (44.4%) | 4 (28.6%) | 4 (28.6%) | 0,127 |
| Unilateral | 16 (26.2%) | 11 (39.3%) | 3 (33.3%) | 4 (28.6%) | 7 (50.0%) |  |
| Bilateral | 9 (14.8%) | 8 (28.6%) | 2 (22.2%) | 6 (42.9%) | 3 (21.4%) |  |
| **TMJ pain attributed to subluxation (ICOP)** |  |  |  |  |  |  |
| No | 61 (100.0%)* | 28 (100.0%)* | 8 (88.9%)* | 7 (50.0%) | 7 (50.0%) | ***<0,001*** |
| Yes | 0 (0.0%) | 0 (0.0%) | 1 (11.1%) | 7 (50.0%)* | 7 (50.0%)* |  |
| **Muscle pain diagnosis (ICOP)** |  |  |  |  |  |  |
| None | 21 (34.4%) | 5 (17.9%) | 2 (22.2%) | 5 (35.7%) | 2 (14.3%) | 0,192 |
| Acute primary orofacial myofascial pain | 3 (4.9%) | 0 (0.0%) | 0 (0.0%) | 0 (0.0%) | 0 (0.0%) |  |
| Frequent chronic primary orofacial myofascial pain without referred pain | 17 (27.9%) | 5 (17.9%) | 0 (0.0%) | 1 (7.1%) | 4 (28.6%) |  |
| Frequent chronic primary orofacial myofascial pain with referred pain | 2 (3.3%) | 2 (7.1%) | 1 (11.1%) | 2 (14.3%) | 1 (7.1%) |  |
| Highly frequent chronic primary orofacial myofascial pain without referred pain | 12 (19.7%) | 11 (39.3%) | 6 (66.7%) | 3 (21.4%) | 4 (28.6%) |  |
| Highly frequent chronic primary orofacial myofascial pain with referred pain | 6 (9.8%) | 5 (17.9%) | 0 (0.0%) | 3 (21.4%) | 3 (21.4%) |  |
| **TMJ pain diagnosis (ICOP)** |  |  |  |  |  |  |
| None | 42 (68.9%)* | 15 (53.6%) | 4 (44.4%) | 6 (42.9%) | 7 (50.0%) | ***0,028*** |
| Acute primary TMJ pain | 3 (4.9%) | 0 (0.0%) | 0 (0.0%) | 0 (0.0%) | 0 (0.0%) |  |
| Frequent chronic primary TMJ pain without referred pain | 3 (4.9%) | 4 (14.3%) | 1 (11.1%) | 1 (7.1%) | 2 (14.3%) |  |
| Frequent chronic primary TMJ pain with referred pain | 2 (3.3%) | 1 (3.6%) | 0 (0.0%) | 1 (7.1%) | 0 (0.0%) |  |
| Highly frequent chronic primary TMJ pain without referred pain | 2 (3.3%) | 3 (10.7%) | 2 (22.2%)* | 0 (0.0%) | 0 (0.0%) |  |
| Highly frequent chronic primary TMJ pain with referred pain | 2 (3.3%) | 3 (10.7%) | 0 (0.0%) | 2 (14.3%)* | 1 (7.1%) |  |
| MJ pain attributed to disc displacement with reduction | 7 (11.5%) | 2 (7.1%) | 1 (11.1%) | 0 (0.0%) | 2 (14.3%)* |  |
| TMJ pain attributed to subluxation | 0 (0.0%) | 0 (0.0%) | 1 (11.1%) | 4 (28.6%)* | 2 (14.3%)* |  |
| **Disc displacement with reduction (DDWR) (DC/TMD)** |  |  |  |  |  |  |
| No | 16 (26.2%) | 9 (32.1%) | 3 (33.3%) | 4 (28.6%) | 2 (14.3%) | 0,916 |
| Unilateral | 36 (59.0%) | 15 (53.6%) | 5 (55.6%) | 7 (50.0%) | 8 (57.1%) |  |
| Bilateral | 9 (14.8%) | 4 (14.3%) | 1 (11.1%) | 3 (21.4%) | 4 (28.6%) |  |
| **DDWR with intermittent locking (DC/TMD)** |  |  |  |  |  |  |
| No | 50 (82.0%) | 25 (89.3%) | 7 (77.8%) | 9 (64.3%) | 10 (71.4%) | 0,341 |
| Yes | 11 (18.0%) | 3 (10.7%) | 2 (22.2%) | 5 (35.7%) | 4 (28.6%) |  |
| **Orofacial pain (VAS)** | 2.54±2.08 | 4.03±2.46* | 2.66±2.50 | 3.53±3.29 | 4.22±2.00* | ***0,024*** |
| Up to 3 | 36 (59.0%) | 9 (32.1%) | 6 (66.7%) | 8 (57.1%) | 4 (28.6%) | 0,052 |
| Over 3 | 25 (41.0%) | 19 (67.9%) | 3 (33.3%) | 6 (42.9%) | 10 (71.4%) |  |
| **Orofacial fatigue (VAS)** | 2.60±1.99 | 3.85±2.37 | 3.70±2.25 | 3.29±2.39 | 3.20±2.28 | 0,129 |
| Up to 3 | 38 (62.3%) | 14 (50.0%) | 4 (44.4%) | 7 (50.0%) | 7 (50.0%) | 0,686 |
| Over 3 | 23 (37.7%) | 14 (50.0%) | 5 (55.6%) | 7 (50.0%) | 7 (50.0%) |  |
| **Orofacial Stiffness (VAS)** | 1.94±2.31 | 2.23±2.92 | 3.48±3.15 | 3.60±2.71 | 4.11±2.53* | ***0,018*** |
| Up to 2 | 38 (62.3%)* | 16 (57.1%)* | 3 (33.3%) | 4 (28.6%) | 3 (21.4%) | ***0,015*** |
| Over 2 | 23 (37.7%) | 12 (42.9%) | 6 (66.7%)* | 10 (71.4%)* | 11 (78.6%)* |  |
| **Orofacial Stiffness (VAS)** | 0.48±1.32 | 1.52±2.77 | 1.41±2.72 | 0.76±1.35 | 0.90±1.34 | 0,149 |
| Up to 1 | 53 (86.9%) | 21 (75.0%) | 7 (77.8%) | 10 (71.4%) | 9 (64.3%) | 0,295 |
| Over 1 | 8 (13.1%) | 7 (25.0%) | 2 (22.2%) | 4 (28.6%) | 5 (35.7%) |  |
| **Orofacial joint instability** **(VAS)** | 1.94±2.89 | 3.61±3.55 | 3.99±3.41 | 4.64±2.50 | 6.48±2.50* | ***<0,001*** |
| Up to 3 | 46 (75.4%)* | 14 (50.0%) | 3 (33.3%) | 3 (21.4%) | 1 (7.1%) | ***<0,001*** |
| Over 3 | 15 (24.6%) | 14 (50.0%)* | 6 (66.7%)* | 11 (78.6%)* | 13 (92.9%)* |  |
| **Generalized joint hypermobility** |  |  |  |  |  |  |
| No | 32 (52.5%) | 9 (32.1%) | 3 (33.3%) | 6 (42.9%) | 5 (35.7%) | 0,382 |
| Yes | 29 (47.5%) | 19 (67.9%) | 6 (66.7%) | 8 (57.1%) | 9 (64.3%) |  |
| **Right TMJ PPT (kgf)** | 1.24±0.51 | 1.25±0.61 | 1.22±0.55 | 1.25±0.42 | 1.25±0.45 | 1,000 |
| Up to 1,1 | 30 (49.2%) | 16 (57.1%) | 4 (44.4%) | 5 (35.7%) | 7 (50.0%) | 0,771 |
| Over 1,1 | 31 (50.8%) | 12 (42.9%) | 5 (55.6%) | 9 (64.3%) | 7 (50.0%) |  |
| **Left TMJ PPT (kgf)** | 1.11±0.37 | 1.09±0.46 | 1.17±0.49 | 1.16±0.42 | 1.16±0.35 | 0,968 |
| Up to 1,1 | 35 (57.4%) | 15 (53.6%) | 5 (55.6%) | 7 (50.0%) | 6 (42.9%) | 0,897 |
| Over 1,1 | 26 (42.6%) | 13 (46.4%) | 4 (44.4%) | 7 (50.0%) | 8 (57.1%) |  |
| **Right masseter PPT (kgf)** | 1.34±0.57 | 1.25±0.57 | 1.31±0.41 | 1.32±0.48 | 1.34±0.44 | 0,969 |
| Up to 1,3 | 31 (50.8%) | 16 (57.1%) | 2 (22.2%) | 5 (35.7%) | 8 (57.1%) | 0,312 |
| Over 1,3 | 30 (49.2%) | 12 (42.9%) | 7 (77.8%) | 9 (64.3%) | 6 (42.9%) |  |
| **Left masseter PPT (kgf)** | 1.30±0.43 | 1.26±0.54 | 1.26±0.52 | 1.37±0.49 | 1.21±0.34 | 0,914 |
| Up to 1,3 | 33 (54.1%) | 19 (67.9%) | 4 (44.4%) | 6 (42.9%) | 6 (42.9%) | 0,420 |
| Over 1,3 | 28 (45.9%) | 9 (32.1%) | 5 (55.6%) | 8 (57.1%) | 8 (57.1%) |  |
| **Right temporalis PPT (kgf)** | 1.56±0.58 | 1.50±0.63 | 1.48±0.50 | 1.42±0.54 | 1.55±0.66 | 0,937 |
| Up to 1,3 | 23 (37.7%) | 14 (50.0%) | 2 (22.2%) | 8 (57.1%) | 6 (42.9%) | 0,411 |
| Over 1,3 | 38 (62.3%) | 14 (50.0%) | 7 (77.8%) | 6 (42.9%) | 8 (57.1%) |  |
| **Left temporalis PPT (kgf)** | 1.44±0.49 | 1.30±0.47 | 1.20±0.22 | 1.30±0.41 | 1.37±0.50 | 0,485 |
| Up to 1,3 | 27 (44.3%) | 15 (53.6%) | 6 (66.7%) | 9 (64.3%) | 7 (50.0%) | 0,548 |
| Over 1,3 | 34 (55.7%) | 13 (46.4%) | 3 (33.3%) | 5 (35.7%) | 7 (50.0%) |  |
| **Pre-fatigue MBF** | 50.09±18.68 | 49.18±14.56 | 47.71±19.59 | 50.07±13.84 | 47.37±16.33 | 0,982 |
| Up to 45 | 29 (47.5%) | 12 (42.9%) | 4 (44.4%) | 6 (42.9%) | 7 (50.0%) | 0,988 |
| Over 45 | 32 (52.5%) | 16 (57.1%) | 5 (55.6%) | 8 (57.1%) | 7 (50.0%) |  |
| **Endurance time** | 119.01±67.47 | 99.88±26.53 | 98.22±25.09 | 107.64±46.47 | 117.67±33.81 | 0,498 |
| Up to 100 | 30 (49.2%) | 14 (50.0%) | 4 (44.4%) | 7 (50.0%) | 6 (42.9%) | 0,991 |
| Over 100 | 31 (50.8%) | 14 (50.0%) | 5 (55.6%) | 7 (50.0%) | 8 (57.1%) |  |
| **Post- fatigue MBF** | 41.10±17.60 | 43.73±15.80 | 39.45±17.04 | 39.52±10.29 | 39.41±13.16 | 0,889 |
| Up to 40 | 35 (57.4%) | 10 (35.7%) | 5 (55.6%) | 8 (57.1%) | 6 (42.9%) | 0,371 |
| Over 40 | 26 (42.6%) | 18 (64.3%) | 4 (44.4%) | 6 (42.9%) | 8 (57.1%) |  |
| **Percentage change in MBF** | 40.49±19.71 | 47.26±21.72 | 41.14±20.30 | 41.85±19.93 | 35.91±15.39 | 0,462 |
| Up to 40 | 32 (52.5%) | 12 (42.9%) | 5 (55.6%) | 9 (64.3%) | 9 (64.3%) | 0,624 |
| Over 40 | 29 (47.5%) | 16 (57.1%) | 4 (44.4%) | 5 (35.7%) | 5 (35.7%) |  |
| **Subjective fatigue (VAS) (post-fatigue)** | 6.68±2.42 | 6.89±2.69 | 6.76±2.85 | 7.65±1.94 | 6.63±2.78 | 0,771 |
| Up to 7 | 29 (47.5%) | 12 (42.9%) | 4 (44.4%) | 4 (28.6%) | 6 (42.9%) | 0,794 |
| Over 7 | 32 (52.5%) | 16 (57.1%) | 5 (55.6%) | 10 (71.4%) | 8 (57.1%) |  |
| **Left TMJ articular capsule** | 1.58±1.80 | 1.42±0.59 | 1.51±0.52 | 1.73±0.91 | 1.54±0.51 | 0,970 |
| Up to 1,4 | 35 (57.4%) | 14 (50.0%) | 4 (44.4%) | 5 (35.7%) | 5 (35.7%) | 0,445 |
| Over 1,4 | 26 (42.6%) | 14 (50.0%) | 5 (55.6%) | 9 (64.3%) | 9 (64.3%) |  |
| **Right TMJ articular capsule** | 1.40±0.61 | 1.36±0.56 | 1.28±0.41 | 1.49±0.50 | 1.35±0.43 | 0,919 |
| Up to 1,4 | 30 (49.2%) | 15 (53.6%) | 5 (55.6%) | 5 (35.7%) | 8 (57.1%) | 0,791 |
| Over 1,4 | 31 (50.8%) | 13 (46.4%) | 4 (44.4%) | 9 (64.3%) | 6 (42.9%) |  |
| **Right masseter (rest)** | 12.80±2.53 | 12.60±2.49 | 14.34±2.42 | 13.40±2.38 | 12.78±1.70 | 0,366 |
| Up to 13 | 33 (54.1%) | 16 (57.1%) | 3 (33.3%) | 6 (42.9%) | 8 (57.1%) | 0,686 |
| Over 13 | 28 (45.9%) | 12 (42.9%) | 6 (66.7%) | 8 (57.1%) | 6 (42.9%) |  |
| **Right masseter (contraction)** | 14.80±2.62 | 14.23±2.35 | 16.57±2.91 | 15.19±2.49 | 15.02±1.98 | 0,187 |
| Up to 15 | 36 (59.0%) | 20 (71.4%) | 3 (33.3%) | 7 (50.0%) | 8 (57.1%) | 0,318 |
| Over 15 | 25 (41.0%) | 8 (28.6%) | 6 (66.7%) | 7 (50.0%) | 6 (42.9%) |  |
| **Left masseter (rest)** | 12.85±2.19 | 12.61±2.30 | 13.93±2.13 | 13.45±2.73 | 12.68±1.82 | 0,501 |
| Up to 13 | 31 (50.8%) | 15 (53.6%) | 5 (55.6%) | 6 (42.9%) | 7 (50.0%) | 0,971 |
| Over 13 | 30 (49.2%) | 13 (46.4%) | 4 (44.4%) | 8 (57.1%) | 7 (50.0%) |  |
| **Left masseter (contraction)** | 14.82±2.45 | 14.34±2.22 | 16.17±2.42 | 15.16±2.57 | 14.96±1.95 | 0,360 |
| Up to 15 | 34 (55.7%) | 16 (57.1%) | 3 (33.3%) | 9 (64.3%) | 7 (50.0%) | 0,663 |
| Over 15 | 27 (44.3%) | 12 (42.9%) | 6 (66.7%) | 5 (35.7%) | 7 (50.0%) |  |
| **Helplessness** | 5.28±3.90 | 6.89±4.04 | 5.89±4.65 | 5.86±4.13 | 8.29±2.89 | 0,089 |
| Up to 6 | 37 (60.7%) | 11 (39.3%) | 5 (55.6%) | 8 (57.1%) | 5 (35.7%) | 0,256 |
| Over 6 | 24 (39.3%) | 17 (60.7%) | 4 (44.4%) | 6 (42.9%) | 9 (64.3%) |  |
| **Magnification** | 3.92±2.87 | 4.75±2.88 | 3.22±2.82 | 4.86±2.88 | 5.43±3.18 | 0,238 |
| Up to 5 | 43 (70.5%) | 18 (64.3%) | 6 (66.7%) | 8 (57.1%) | 9 (64.3%) | 0,897 |
| Over 5 | 18 (29.5%) | 10 (35.7%) | 3 (33.3%) | 6 (42.9%) | 5 (35.7%) |  |
| **Rumination** | 4.82±4.13 | 6.39±5.07 | 4.56±4.28 | 6.07±4.34 | 6.50±3.44 | 0,394 |
| Up to 5 | 39 (63.9%) | 10 (35.7%) | 6 (66.7%) | 9 (64.3%) | 6 (42.9%) | 0,092 |
| Over 5 | 22 (36.1%) | 18 (64.3%) | 3 (33.3%) | 5 (35.7%) | 8 (57.1%) |  |
| **Total catastrophizing** | 13.85±10.07 | 18.04±11.21 | 13.67±11.37 | 16.57±9.99 | 20.21±8.45 | 0,167 |
| Up to 15 | 36 (59.0%) | 9 (32.1%) | 6 (66.7%) | 6 (42.9%) | 5 (35.7%) | 0,093 |
| Over 15 | 25 (41.0%) | 19 (67.9%) | 3 (33.3%) | 8 (57.1%) | 9 (64.3%) |  |
| **Mandibular kinesiophobia** | 25.41±6.22 | 29.93±4.76* | 30.00±5.15* | 29.14±5.40* | 31.71±3.93* | ***<0,001*** |
| Up to 30 | 47 (77.0%)* | 14 (50.0%) | 4 (44.4%) | 9 (64.3%) | 5 (35.7%) | ***0,011*** |
| Over 30 | 14 (23.0%) | 14 (50.0%) | 5 (55.6%) | 5 (35.7%) | 9 (64.3%)* |  |
| **Hypervigilance** | 41.80±14.49 | 44.07±14.89 | 49.22±14.61 | 43.71±13.98 | 50.00±12.93 | 0,288 |
| Up to 40 | 30 (49.2%) | 11 (39.3%) | 2 (22.2%) | 7 (50.0%) | 3 (21.4%) | 0,230 |
| Over 40 | 31 (50.8%) | 17 (60.7%) | 7 (77.8%) | 7 (50.0%) | 11 (78.6%) |  |
| **JFLS score** | 32.25±24.81 | 48.86±30.56 | 43.00±17.92 | 53.36±36.70* | 56.50±29.45* | ***0,006*** |
| Up to 35 | 38 (62.3%) | 14 (50.0%) | 3 (33.3%) | 6 (42.9%) | 4 (28.6%) | 0,115 |
| Over 35 | 23 (37.7%) | 14 (50.0%) | 6 (66.7%) | 8 (57.1%) | 10 (71.4%) |  |
| **JFLS items 7 and 12** | 8.79±5.45 | 11.39±4.13 | 12.00±4.74 | 12.07±5.43 | 15.07±3.34* | ***<0,001*** |
| Up to 10 | 37 (60.7%)* | 13 (46.4%)* | 4 (44.4%)* | 6 (42.9%)* | 2 (14.3%) | ***0,034*** |
| Over 10 | 24 (39.3%) | 15 (53.6%) | 5 (55.6%) | 8 (57.1%) | 12 (85.7%)* |  |

Table 1. Bivariate associations between the frequency of open-locking episodes and clinical, functional, and psychosocial variables. p<0.05, Fisher’s exact test or Pearson’s chi-square test (n, %); p<0.05, Kruskal–Wallis test with Dunn’s post hoc (mean±SD).
