# Supplementary material for: Predictive Value of Diagnostic Methods for TMJ Hypermobility in the Associated Clinical and Functional Features of Temporomandibular Disorders: A Regression Study
Source: J Oral Rehabil. 2025 Nov 27;53(3):673–84. doi: 10.1111/joor.70120 (PMC12902195; doi:10.1111/joor.70120)
Supplement: Supplementary file 2 — Table S2: Bivariate associations between the right and left TMJ angle and clinical, functional and psychosocial variables. *p < 0.05, Fisher's exact test or Pearson's chi‐square test (n, %); *p < 0.05, Mann–Whitney test (mean ± SD). [file JOOR-53-673-s004.docx]

|  | **Right TMJ angle** | |  | **Left TMJ angle** | |  |
| --- | --- | --- | --- | --- | --- | --- |
|  | **Up to 280º** | **Over 280º** | **p-**  **value** | **Up to 280º** | **Over 280º** | **p-value** |
| **Sex** |  |  |  |  |  |  |
| Male | 11 (21.6%) | 21 (28.0%) | 0,416 | 14 (25.5%) | 18 (25.4%) | 0,990 |
| Female | 40 (78.4%) | 54 (72.0%) |  | 41 (74.5%) | 53 (74.6%) |  |
| **Age** | 26.04±4.94 | 27.89±6.07 | 0,073 | 26.47±4.95 | 27.66±6.19 | 0,247 |
| Up to 25 | 26 (51.0%) | 32 (42.7%) | 0,358 | 25 (45.5%) | 33 (46.5%) | 0,909 |
| Over 25 | 25 (49.0%) | 43 (57.3%) |  | 30 (54.5%) | 38 (53.5%) |  |
| **Open-locking episodes** |  |  |  |  |  |  |
| Never | 27 (52.9%) | 34 (45.3%) | 0,460 | 28 (50.9%) | 33 (46.5%) | 0,974 |
| Once in a lifetime | 10 (19.6%) | 18 (24.0%) |  | 12 (21.8%) | 16 (22.5%) |  |
| Once a year | 5 (9.8%) | 4 (5.3%) |  | 4 (7.3%) | 5 (7.0%) |  |
| Once a month | 6 (11.8%) | 8 (10.7%) |  | 5 (9.1%) | 9 (12.7%) |  |
| More than once a month | 3 (5.9%) | 11 (14.7%) |  | 6 (10.9%) | 8 (11.3%) |  |
| **Pain-free maximum mouth opening** | 37.92±11.16 | 45.03±12.05 | ***<0,001*** | 38.29±11.45 | 45.14±11.93 | ***<0,001*** |
| Up to 40 | 27 (52.9%) | 25 (33.3%) | ***0,028*** | 29 (52.7%) | 23 (32.4%) | ***0,021*** |
| Over 40 | 24 (47.1%) | 50 (66.7%) |  | 26 (47.3%) | 48 (67.6%) |  |
| **Unassisted maximum mouth opening** | 51.84±6.29 | 57.21±7.09 | ***<0,001*** | 52.53±6.60 | 56.99±7.18 | ***<0,001*** |
| Up to 55 | 38 (74.5%)* | 29 (38.7%) | ***<0,001*** | 35 (63.6%)* | 32 (45.1%) | ***0,021*** |
| Over 55 | 13 (25.5%) | 46 (61.3%)* |  | 20 (36.4%) | 39 (54.9%)* |  |
| **Assisted maximum mouth opening** | 55.18±6.01 | 60.44±6.09 | ***<0,001*** | 55.53±6.10 | 60.46±6.13 | ***<0,001*** |
| Up to 55 | 27 (52.9%)* | 16 (21.3%) | ***<0,001*** | 28 (50.9%)* | 15 (21.1%) | ***0,038*** |
| Over 55 | 24 (47.1%) | 59 (78.7%)* |  | 27 (49.1%) | 56 (78.9%)* |  |
| **Lateral condylar jump** |  |  |  |  |  |  |
| No | 15 (29.4%)* | 10 (13.3%) | ***0,026*** | 18 (32.7%)* | 7 (9.9%) | ***<0,001*** |
| Yes | 36 (70.6%) | 65 (86.7%)* |  | 37 (67.3%) | 64 (90.1%)* |  |
| **Midline deviation during opening** |  |  |  |  |  |  |
| No | 13 (25.5%)* | 6 (8.0%) | ***0,007*** | 14 (25.5%)* | 5 (7.0%) | ***0,004*** |
| Yes | 38 (74.5%) | 69 (92.0%)* |  | 41 (74.5%) | 66 (93.0%)* |  |
| **Terminal click** |  |  |  |  |  |  |
| No | 49 (96.1%)* | 61 (81.3%) | ***0,015*** | 53 (96.4%)* | 57 (80.3%) | ***0,007*** |
| Yes | 2 (3.9%) | 14 (18.7%)* |  | 2 (3.6%) | 14 (19.7%)* |  |
| **Subluxation diagnosis (DC/TMD)** |  |  |  |  |  |  |
| No | 44 (86.3%) | 57 (76.0%) | 0,156 | 47 (85.5%) | 54 (76.1%) | 0,190 |
| Yes | 7 (13.7%) | 18 (24.0%) |  | 8 (14.5%) | 17 (23.9%) |  |
| **Muscular TMD diagnosis (DC/TMD)** |  |  |  |  |  |  |
| None | 12 (23.5%) | 21 (28.0%) | 0,694 | 11 (20.0%) | 22 (31.0%) | 0,492 |
| Local myalgia | 10 (19.6%) | 18 (24.0%) |  | 12 (21.8%) | 16 (22.5%) |  |
| Myofascial pain | 17 (33.3%) | 18 (24.0%) |  | 18 (32.7%) | 17 (23.9%) |  |
| Myofascial pain with referral | 12 (23.5%) | 18 (24.0%) |  | 14 (25.5%) | 16 (22.5%) |  |
| **Headache attributed to TMD** |  |  |  |  |  |  |
| No | 29 (56.9%) | 39 (52.0%) | 0,591 | 27 (49.1%) | 41 (57.7%) | 0,334 |
| Yes | 22 (43.1%) | 36 (48.0%) |  | 28 (50.9%) | 30 (42.3%) |  |
| **Arthralgia** |  |  |  |  |  |  |
| No | 17 (33.3%) | 40 (53.3%) | 0,072 | 20 (36.4%) | 37 (52.1%) | 0,178 |
| Unilateral | 19 (37.3%) | 22 (29.3%) |  | 22 (40.0%) | 19 (26.8%) |  |
| Bilateral | 15 (29.4%) | 13 (17.3%) |  | 13 (23.6%) | 15 (21.1%) |  |
| **TMJ pain attributed to subluxation (ICOP)** |  |  |  |  |  |  |
| No | 45 (88.2%) | 66 (88.0%) | 0,968 | 48 (87.3%) | 63 (88.7%) | 0,802 |
| Yes | 6 (11.8%) | 9 (12.0%) |  | 7 (12.7%) | 8 (11.3%) |  |
| **Muscle pain diagnosis (ICOP)** |  |  |  |  |  |  |
| None | 12 (23.5%) | 23 (30.7%) | 0,650 | 11 (20.0%) | 24 (33.8%) | 0,594 |
| Acute primary orofacial myofascial pain | 2 (3.9%) | 1 (1.3%) |  | 1 (1.8%) | 2 (2.8%) |  |
| Frequent chronic primary orofacial myofascial pain without referred pain | 10 (19.6%) | 17 (22.7%) |  | 12 (21.8%) | 15 (21.1%) |  |
| Frequent chronic primary orofacial myofascial pain with referred pain | 2 (3.9%) | 6 (8.0%) |  | 4 (7.3%) | 4 (5.6%) |  |
| Highly frequent chronic primary orofacial myofascial pain without referred pain | 17 (33.3%) | 19 (25.3%) |  | 19 (34.5%) | 17 (23.9%) |  |
| Highly frequent chronic primary orofacial myofascial pain with referred pain | 8 (15.7%) | 9 (12.0%) |  | 8 (14.5%) | 9 (12.7%) |  |
| **TMJ pain diagnosis (ICOP)** |  |  |  |  |  |  |
| None | 22 (43.1%) | 52 (69.3%) | 0,062 | 29 (52.7%) | 45 (63.4%) | 0,636 |
| Acute primary TMJ pain | 3 (5.9%) | 0 (0.0%) |  | 2 (3.6%) | 1 (1.4%) |  |
| Frequent chronic primary TMJ pain without referred pain | 5 (9.8%) | 6 (8.0%) |  | 6 (10.9%) | 5 (7.0%) |  |
| Frequent chronic primary TMJ pain with referred pain | 2 (3.9%) | 2 (2.7%) |  | 3 (5.5%) | 1 (1.4%) |  |
| Highly frequent chronic primary TMJ pain without referred pain | 4 (7.8%) | 3 (4.0%) |  | 3 (5.5%) | 4 (5.6%) |  |
| Highly frequent chronic primary TMJ pain with referred pain | 6 (11.8%) | 2 (2.7%) |  | 5 (9.1%) | 3 (4.2%) |  |
| MJ pain attributed to disc displacement with reduction | 6 (11.8%) | 6 (8.0%) |  | 5 (9.1%) | 7 (9.9%) |  |
| TMJ pain attributed to subluxation | 3 (5.9%) | 4 (5.3%) |  | 2 (3.6%) | 5 (7.0%) |  |
| **Disc displacement with reduction (DDWR) (DC/TMD)** |  |  |  |  |  |  |
| No | 13 (25.5%) | 21 (28.0%) | 0,940 | 15 (27.3%) | 19 (26.8%) | 0,996 |
| Unilateral | 29 (56.9%) | 42 (56.0%) |  | 31 (56.4%) | 40 (56.3%) |  |
| Bilateral | 9 (17.6%) | 12 (16.0%) |  | 9 (16.4%) | 12 (16.9%) |  |
| **DDWR with intermittent locking (DC/TMD)** |  |  |  |  |  |  |
| No | 40 (78.4%) | 61 (81.3%) | 0,688 | 47 (85.5%) | 54 (76.1%) | 0,190 |
| Yes | 11 (21.6%) | 14 (18.7%) |  | 8 (14.5%) | 17 (23.9%) |  |
| **Orofacial pain (VAS)** | 3.71±2.54 | 2.81±2.27 | ***0,039*** | 3.68±2.36 | 2.78±2.40 | ***0,038*** |
| Up to 3 | 21 (41.2%) | 42 (56.0%) | 0,102 | 24 (43.6%) | 39 (54.9%) | 0,209 |
| Over 3 | 30 (58.8%) | 33 (44.0%) |  | 31 (56.4%) | 32 (45.1%) |  |
| **Orofacial fatigue (VAS)** | 3.16±2.05 | 3.06±2.31 | 0,798 | 3.08±2.08 | 3.12±2.31 | 0,917 |
| Up to 3 | 27 (52.9%) | 43 (57.3%) | 0,626 | 31 (56.4%) | 39 (54.9%) | 0,872 |
| Over 3 | 24 (47.1%) | 32 (42.7%) |  | 24 (43.6%) | 32 (45.1%) |  |
| **Orofacial Stiffness (VAS)** | 2.56±2.69 | 2.52±2.67 | 0,934 | 2.33±2.54 | 2.70±2.78 | 0,440 |
| Up to 2 | 25 (49.0%) | 39 (52.0%) | 0,743 | 28 (50.9%) | 36 (50.7%) | 0,982 |
| Over 2 | 26 (51.0%) | 36 (48.0%) |  | 27 (49.1%) | 35 (49.3%) |  |
| **Orofacial Stiffness (VAS)** | 0.83±1.83 | 0.87±1.93 | 0,917 | 0.75±1.71 | 0.93±2.01 | 0,594 |
| Up to 1 | 40 (78.4%) | 60 (80.0%) | 0,831 | 44 (80.0%) | 56 (78.9%) | 0,877 |
| Over 1 | 11 (21.6%) | 15 (20.0%) |  | 11 (20.0%) | 15 (21.1%) |  |
| **Orofacial joint instability** **(VAS)** | 3.38±3.55 | 3.18±3.18 | 0,736 | 2.89±3.32 | 3.55±3.32 | 0,269 |
| Up to 3 | 27 (52.9%) | 40 (53.3%) | 0,965 | 32 (58.2%) | 35 (49.3%) | 0,321 |
| Over 3 | 24 (47.1%) | 35 (46.7%) |  | 23 (41.8%) | 36 (50.7%) |  |
| **Generalized joint hypermobility** |  |  |  |  |  |  |
| No | 22 (43.1%) | 33 (44.0%) | 0,924 | 24 (43.6%) | 31 (43.7%) | 0,998 |
| Yes | 29 (56.9%) | 42 (56.0%) |  | 31 (56.4%) | 40 (56.3%) |  |
| **Right TMJ PPT (kgf)** | 1.17±0.47 | 1.30±0.54 | 0,164 | 1.16±0.45 | 1.31±0.55 | 0,105 |
| Up to 1,1 | 30 (58.8%) | 32 (42.7%) | 0,075 | 33 (60.0%)* | 29 (40.8%) | ***0,033*** |
| Over 1,1 | 21 (41.2%) | 43 (57.3%) |  | 22 (40.0%) | 42 (59.2%)* |  |
| **Right TMJ PPT (kgf)** | 1.05±0.37 | 1.17±0.41 | 0,106 | 1.05±0.37 | 1.18±0.41 | 0,084 |
| Up to 1,1 | 32 (62.7%) | 36 (48.0%) | 0,103 | 35 (63.6%) | 33 (46.5%) | 0,055 |
| Over 1,1 | 19 (37.3%) | 39 (52.0%) |  | 20 (36.4%) | 38 (53.5%) |  |
| **Right masseter PPT (kgf)** | 1.26±0.49 | 1.35±0.56 | 0,371 | 1.22±0.50 | 1.39±0.54 | 0,078 |
| Up to 1,3 | 27 (52.9%) | 35 (46.7%) | 0,489 | 32 (58.2%) | 30 (42.3%) | 0,076 |
| Over 1,3 | 24 (47.1%) | 40 (53.3%) |  | 23 (41.8%) | 41 (57.7%) |  |
| **Left masseter PPT (kgf)** | 1.23±0.42 | 1.32±0.48 | 0,273 | 1.21±0.43 | 1.34±0.47 | 0,112 |
| Up to 1,3 | 31 (60.8%) | 37 (49.3%) | 0,206 | 33 (60.0%) | 35 (49.3%) | 0,232 |
| Over 1,3 | 20 (39.2%) | 38 (50.7%) |  | 22 (40.0%) | 36 (50.7%) |  |
| **Right temporalis PPT (kgf)** | 1.46±0.56 | 1.57±0.60 | 0,305 | 1.47±0.59 | 1.57±0.57 | 0,389 |
| Up to 1,3 | 24 (47.1%) | 29 (38.7%) | 0,349 | 25 (45.5%) | 28 (39.4%) | 0,497 |
| Over 1,3 | 27 (52.9%) | 46 (61.3%) |  | 30 (54.5%) | 43 (60.6%) |  |
| **Left temporalis PPT (kgf)** | 1.29±0.41 | 1.42±0.49 | 0,100 | 1.30±0.45 | 1.42±0.47 | 0,165 |
| Up to 1,3 | 31 (60.8%) | 33 (44.0%) | 0,064 | 30 (54.5%) | 34 (47.9%) | 0,458 |
| Over 1,3 | 20 (39.2%) | 42 (56.0%) |  | 25 (45.5%) | 37 (52.1%) |  |
| **Pre-fatigue MBF** | 47.72±16.87 | 50.57±16.98 | 0,356 | 46.70±17.98 | 51.52±15.87 | 0,114 |
| Up to 45 | 26 (51.0%) | 32 (42.7%) | 0,358 | 32 (58.2%)* | 26 (36.6%) | ***0,001*** |
| Over 45 | 25 (49.0%) | 43 (57.3%) |  | 23 (41.8%) | 45 (63.4%)* |  |
| **Endurance time** | 111.28±67.51 | 112.26±40.56 | 0,920 | 112.80±64.08 | 111.13±42.73 | 0,862 |
| Up to 100 | 26 (51.0%) | 35 (46.7%) | 0,634 | 26 (47.3%) | 35 (49.3%) | 0,822 |
| Over 100 | 25 (49.0%) | 40 (53.3%) |  | 29 (52.7%) | 36 (50.7%) |  |
| **Post- fatigue MBF** | 38.60±15.87 | 42.97±15.77 | 0,130 | 38.50±16.35 | 43.30±15.31 | 0,093 |
| Up to 40 | 29 (56.9%) | 35 (46.7%) | 0,261 | 31 (56.4%) | 33 (46.5%) | 0,271 |
| Over 40 | 22 (43.1%) | 40 (53.3%) |  | 24 (43.6%) | 38 (53.5%) |  |
| **Percentage change in MBF** | 41.32±21.69 | 41.93±18.56 | 0,866 | 40.76±22.65 | 42.40±17.41 | 0,647 |
| Up to 40 | 29 (56.9%) | 38 (50.7%) | 0,494 | 31 (56.4%) | 36 (50.7%) | 0,528 |
| Over 40 | 22 (43.1%) | 37 (49.3%) |  | 24 (43.6%) | 35 (49.3%) |  |
| **Subjective fatigue (VAS) (post-fatigue)** | 6.74±2.59 | 6.90±2.43 | 0,730 | 6.70±2.41 | 6.94±2.55 | 0,585 |
| Up to 7 | 24 (47.1%) | 31 (41.3%) | 0,525 | 27 (49.1%) | 28 (39.4%) | 0,279 |
| Over 7 | 27 (52.9%) | 44 (58.7%) |  | 28 (50.9%) | 43 (60.6%) |  |
| **Left TMJ articular capsule** | 1.79±1.91 | 1.39±0.67 | 0,094 | 1.49±0.61 | 1.60±1.70 | 0,649 |
| Up to 1,4 | 21 (41.2%) | 42 (56.0%) | 0,102 | 25 (45.5%) | 38 (53.5%) | 0,369 |
| Over 1,4 | 30 (58.8%) | 33 (44.0%) |  | 30 (54.5%) | 33 (46.5%) |  |
| **Right TMJ articular capsule** | 1.52±0.52 | 1.30±0.56 | ***0,032*** | 1.44±0.53 | 1.35±0.57 | 0,355 |
| Up to 1,4 | 20 (39.2%) | 43 (57.3%)* | ***0,046*** | 25 (45.5%) | 38 (53.5%) | 0,369 |
| Over 1,4 | 31 (60.8%)* | 32 (42.7%) |  | 30 (54.5%) | 33 (46.5%) |  |
| **Right masseter (rest)** | 13.08±2.14 | 12.83±2.61 | 0,558 | 12.98±2.15 | 12.89±2.63 | 0,843 |
| Up to 13 | 24 (47.1%) | 42 (56.0%) | 0,324 | 28 (50.9%) | 38 (53.5%) | 0,771 |
| Over 13 | 27 (52.9%) | 33 (44.0%) |  | 27 (49.1%) | 33 (46.5%) |  |
| **Right masseter (contraction)** | 14.87±2.13 | 14.86±2.79 | 0,983 | 14.70±2.27 | 14.99±2.73 | 0,518 |
| Up to 15 | 31 (60.8%) | 43 (57.3%) | 0,699 | 34 (61.8%) | 40 (56.3%) | 0,535 |
| Over 15 | 20 (39.2%) | 32 (42.7%) |  | 21 (38.2%) | 31 (43.7%) |  |
| **Left masseter (rest)** | 13.01±1.67 | 12.86±2.56 | 0,719 | 12.74±1.99 | 13.06±2.41 | 0,429 |
| Up to 13 | 25 (49.0%) | 39 (52.0%) | 0,743 | 28 (50.9%) | 36 (50.7%) | 0,982 |
| Over 13 | 26 (51.0%) | 36 (48.0%) |  | 27 (49.1%) | 35 (49.3%) |  |
| **Left masseter (contraction)** | 14.76±1.92 | 14.93±2.64 | 0,697 | 14.56±2.35 | 15.10±2.37 | 0,202 |
| Up to 15 | 32 (62.7%) | 37 (49.3%) | 0,138 | 34 (61.8%) | 35 (49.3%) | 0,161 |
| Over 15 | 19 (37.3%) | 38 (50.7%) |  | 21 (38.2%) | 36 (50.7%) |  |
| **Helplessness** | 6.57±4.16 | 5.75±3.85 | 0,257 | 6.40±4.00 | 5.83±3.98 | 0,429 |
| Up to 6 | 23 (45.1%) | 43 (57.3%) | 0,177 | 26 (47.3%) | 40 (56.3%) | 0,312 |
| Over 6 | 28 (54.9%) | 32 (42.7%) |  | 29 (52.7%) | 31 (43.7%) |  |
| **Magnification** | 4.73±3.13 | 4.05±2.76 | 0,206 | 4.71±3.12 | 4.03±2.74 | 0,195 |
| Up to 5 | 28 (54.9%) | 56 (74.7%)* | ***0,021*** | 32 (58.2%) | 52 (73.2%) | 0,075 |
| Over 5 | 23 (45.1%)* | 19 (25.3%) |  | 23 (41.8%) | 19 (26.8%) |  |
| **Rumination** | 5.59±4.36 | 5.40±4.32 | 0,812 | 5.53±4.25 | 5.44±4.41 | 0,908 |
| Up to 5 | 26 (51.0%) | 44 (58.7%) | 0,394 | 28 (50.9%) | 42 (59.2%) | 0,356 |
| Over 5 | 25 (49.0%) | 31 (41.3%) |  | 27 (49.1%) | 29 (40.8%) |  |
| **Total catastrophizing score** | 16.63±10.65 | 15.20±10.20 | 0,450 | 16.40±10.31 | 15.30±10.46 | 0,555 |
| Up to 15 | 21 (41.2%) | 41 (54.7%) | 0,137 | 25 (45.5%) | 37 (52.1%) | 0,458 |
| Over 15 | 30 (58.8%) | 34 (45.3%) |  | 30 (54.5%) | 34 (47.9%) |  |
| **Mandibular kinesiophobia** | 28.75±6.53 | 27.25±5.56 | 0,171 | 28.11±6.40 | 27.66±5.69 | 0,679 |
| Up to 30 | 29 (56.9%) | 50 (66.7%) | 0,264 | 35 (63.6%) | 44 (62.0%) | 0,848 |
| Over 30 | 22 (43.1%) | 25 (33.3%) |  | 20 (36.4%) | 27 (38.0%) |  |
| **Hypervigilance** | 48.39±14.68 | 40.95±13.55 | ***0,004*** | 44.80±14.61 | 43.31±14.37 | 0,568 |
| Up to 40 | 14 (27.5%) | 39 (52.0%)* | ***0,006*** | 20 (36.4%) | 33 (46.5%) | 0,254 |
| Over 40 | 37 (72.5%)* | 36 (48.0%) |  | 35 (63.6%) | 38 (53.5%) |  |
| **JFLS score** | 48.33±29.27 | 37.27±28.19 | ***0,035*** | 43.64±31.36 | 40.28±27.23 | 0,522 |
| Up to 35 | 21 (41.2%) | 44 (58.7%) | 0,054 | 28 (50.9%) | 37 (52.1%) | 0,893 |
| Over 35 | 30 (58.8%) | 31 (41.3%) |  | 27 (49.1%) | 34 (47.9%) |  |
| **JFLS items 7 and 12** | 11.69±4.99 | 9.96±5.41 | 0,072 | 10.38±5.57 | 10.87±5.09 | 0,607 |
| Up to 10 | 23 (45.1%) | 39 (52.0%) | 0,447 | 29 (52.7%) | 33 (46.5%) | 0,487 |
| Over 10 | 28 (54.9%) | 36 (48.0%) |  | 26 (47.3%) | 38 (53.5%) |  |

Table 2. Bivariate associations between the right and left TMJ angle and clinical, functional, and psychosocial variables. *p < 0.05, Fisher’s exact test or Pearson’s chi-square test (n, %); *p < 0.05, Mann–Whitney test (mean ± SD).
