# Supplementary material for: Predictive Value of Diagnostic Methods for TMJ Hypermobility in the Associated Clinical and Functional Features of Temporomandibular Disorders: A Regression Study
Source: J Oral Rehabil. 2025 Nov 27;53(3):673–84. doi: 10.1111/joor.70120 (PMC12902195; doi:10.1111/joor.70120)
Supplement: Supplementary file 3 — Table S3: Bivariate analysis considering maximum assisted and unassisted mouth opening as predictors of clinical, functional and psychosocial outcomes. *p < 0.05, Fisher's exact test or Pearson's chi‐square test (n, %); *p < 0.05, Mann–Whitney test (mean ± SD). [file JOOR-53-673-s003.docx]

|  | **Maximum unassisted mouth opening** | | **p-value** | **Maximum assisted mouth opening** | | **p- value** |
| --- | --- | --- | --- | --- | --- | --- |
|  | **Up to 55** | **Over 55** |  | **Up to 55** | **Over 55** |  |
| **Sex** |  |  |  |  |  |  |
| Male | 12 (17.9%) | 20 (33.9%)* | ***0,040*** | 7 (16.3%) | 25 (30.1%) | 0,091 |
| Female | 55 (82.1%)* | 39 (66.1%) |  | 36 (83.7%) | 58 (69.9%) |  |
| **Age** | 26.93±5.57 | 27.39±5.87 | 0,650 | 27.37±5.63 | 27.02±5.76 | 0,746 |
| Up to 25 | 31 (46.3%) | 27 (45.8%) | 0,955 | 19 (44.2%) | 39 (47.0%) | 0,765 |
| Over 25 | 36 (53.7%) | 32 (54.2%) |  | 24 (55.8%) | 44 (53.0%) |  |
| **Right TMJ angle** | 266.44±39.30 | 283.38±10.30 | ***0,002*** | 262.13±41.38 | 280.71±20.78 | ***0,001*** |
| Up to 280º | 38 (56.7%)* | 13 (22.0%) | ***<0,001*** | 27 (62.8%)* | 24 (28.9%) | ***<0,001*** |
| Over 280º | 29 (43.3%) | 46 (78.0%)* |  | 16 (37.2%) | 59 (71.1%)* |  |
| **Left TMJ angle** | 267.39±38.79 | 282.13±12.59 | ***0,006*** | 260.66±45.76 | 281.36±13.63 | ***<0,001*** |
| Up to 280º | 35 (52.2%)* | 20 (33.9%) | ***0,038*** | 28 (65.1%)* | 27 (32.5%) | ***<0,001*** |
| Over 280º | 32 (47.8%) | 39 (66.1%)* |  | 15 (34.9%) | 56 (67.5%)* |  |
| **Open-locking episodes** |  |  |  |  |  |  |
| Never | 34 (50.7%) | 27 (45.8%) | 0,654 | 27 (62.8%)* | 34 (41.0%) | ***0,024*** |
| Once in a lifetime | 14 (20.9%) | 14 (23.7%) |  | 11 (25.6%) | 17 (20.5%) |  |
| Once a year | 3 (4.5%) | 6 (10.2%) |  | 0 (0.0%) | 9 (10.8%)* |  |
| Once a month | 9 (13.4%) | 5 (8.5%) |  | 2 (4.7%) | 12 (14.5%)* |  |
| More than once a month | 7 (10.4%) | 7 (11.9%) |  | 3 (7.0%) | 11 (13.3%)* |  |
| **Pain-free maximum mouth opening** | 34.99±8.73 | 50.29±10.28 | ***<0,001*** | 35.40±8.92 | 45.65±12.19 | ***<0,001*** |
| Up to 40 | 43 (64.2%)* | 9 (15.3%) | ***<0,001*** | 27 (62.8%)* | 25 (30.1%) | ***<0,001*** |
| Over 40 | 24 (35.8%) | 50 (84.7%)* |  | 16 (37.2%) | 58 (69.9%)* |  |
| **Lateral condylar jump** |  |  |  |  |  |  |
| No | 19 (28.4%)* | 6 (10.2%) | ***0,011*** | 16 (37.2%)* | 9 (10.8%) | ***<0,001*** |
| Yes | 48 (71.6%) | 53 (89.8%)* |  | 27 (62.8%) | 74 (89.2%)* |  |
| **Midline deviation during opening** |  |  |  |  |  |  |
| No | 15 (22.4%)* | 4 (6.8%) | ***0,015*** | 12 (27.9%)* | 7 (8.4%) | ***0,004*** |
| Yes | 52 (77.6%) | 55 (93.2%)* |  | 31 (72.1%) | 76 (91.6%)* |  |
| **Terminal click** |  |  |  |  |  |  |
| No | 59 (88.1%) | 51 (86.4%) | 0,785 | 40 (93.0%) | 70 (84.3%) | 0,165 |
| Yes | 8 (11.9%) | 8 (13.6%) |  | 3 (7.0%) | 13 (15.7%) |  |
| **Subluxation diagnosis (DC/TMD)** |  |  |  |  |  |  |
| No | 54 (80.6%) | 47 (79.7%) | 0,895 | 39 (90.7%)* | 62 (74.7%) | ***0,033*** |
| Yes | 13 (19.4%) | 12 (20.3%) |  | 4 (9.3%) | 21 (25.3%)* |  |
| **Muscular TMD diagnosis (DC/TMD)** |  |  |  |  |  |  |
| None | 11 (16.4%) | 22 (37.3%) | 0,070 | 10 (23.3%) | 23 (27.7%) | 0,941 |
| Local myalgia | 17 (25.4%) | 11 (18.6%) |  | 10 (23.3%) | 18 (21.7%) |  |
| Myofascial pain | 21 (31.3%) | 14 (23.7%) |  | 13 (30.2%) | 22 (26.5%) |  |
| Myofascial pain with referral | 18 (26.9%) | 12 (20.3%) |  | 10 (23.3%) | 20 (24.1%) |  |
| **Headache attributed to TMD** |  |  |  |  |  |  |
| No | 34 (50.7%) | 34 (57.6%) | 0,439 | 22 (51.2%) | 46 (55.4%) | 0,649 |
| Yes | 33 (49.3%) | 25 (42.4%) |  | 21 (48.8%) | 37 (44.6%) |  |
| **Arthralgia** |  |  |  |  |  |  |
| No | 27 (40.3%) | 30 (50.8%) | 0,460 | 20 (46.5%) | 37 (44.6%) | 0,964 |
| Unilateral | 23 (34.3%) | 18 (30.5%) |  | 14 (32.6%) | 27 (32.5%) |  |
| Bilateral | 17 (25.4%) | 11 (18.6%) |  | 9 (20.9%) | 19 (22.9%) |  |
| **TMJ pain attributed to subluxation (ICOP)** |  |  |  |  |  |  |
| No | 57 (85.1%) | 54 (91.5%) | 0,265 | 41 (95.3%) | 70 (84.3%) | 0,070 |
| Yes | 10 (14.9%) | 5 (8.5%) |  | 2 (4.7%) | 13 (15.7%) |  |
| **Muscle pain diagnosis (ICOP)** |  |  |  |  |  |  |
| None | 12 (17.9%) | 23 (39.0%) | 0,104 | 10 (23.3%) | 25 (30.1%) | 0,957 |
| Acute primary orofacial myofascial pain | 3 (4.5%) | 0 (0.0%) |  | 1 (2.3%) | 2 (2.4%) |  |
| Frequent chronic primary orofacial myofascial pain without referred pain | 15 (22.4%) | 12 (20.3%) |  | 11 (25.6%) | 16 (19.3%) |  |
| Frequent chronic primary orofacial myofascial pain with referred pain | 5 (7.5%) | 3 (5.1%) |  | 3 (7.0%) | 5 (6.0%) |  |
| Highly frequent chronic primary orofacial myofascial pain without referred pain | 22 (32.8%) | 14 (23.7%) |  | 12 (27.9%) | 24 (28.9%) |  |
| Highly frequent chronic primary orofacial myofascial pain with referred pain | 10 (14.9%) | 7 (11.9%) |  | 6 (14.0%) | 11 (13.3%) |  |
| **TMJ pain diagnosis (ICOP)** |  |  |  |  |  |  |
| None | 35 (52.2%) | 39 (66.1%) | 0,579 | 25 (58.1%) | 49 (59.0%) | 0,541 |
| Acute primary TMJ pain | 3 (4.5%) | 0 (0.0%) |  | 2 (4.7%) | 1 (1.2%) |  |
| Frequent chronic primary TMJ pain without referred pain | 7 (10.4%) | 4 (6.8%) |  | 3 (7.0%) | 8 (9.6%) |  |
| Frequent chronic primary TMJ pain with referred pain | 3 (4.5%) | 1 (1.7%) |  | 3 (7.0%) | 1 (1.2%) |  |
| Highly frequent chronic primary TMJ pain without referred pain | 3 (4.5%) | 4 (6.8%) |  | 2 (4.7%) | 5 (6.0%) |  |
| Highly frequent chronic primary TMJ pain with referred pain | 5 (7.5%) | 3 (5.1%) |  | 3 (7.0%) | 5 (6.0%) |  |
| MJ pain attributed to disc displacement with reduction | 7 (10.4%) | 5 (8.5%) |  | 4 (9.3%) | 8 (9.6%) |  |
| TMJ pain attributed to subluxation | 4 (6.0%) | 3 (5.1%) |  | 1 (2.3%) | 6 (7.2%) |  |
| **Disc displacement with reduction (DDWR) (DC/TMD)** |  |  |  |  |  |  |
| No | 19 (28.4%) | 15 (25.4%) | 0,934 | 12 (27.9%) | 22 (26.5%) | 0,985 |
| Unilateral | 37 (55.2%) | 34 (57.6%) |  | 24 (55.8%) | 47 (56.6%) |  |
| Bilateral | 11 (16.4%) | 10 (16.9%) |  | 7 (16.3%) | 14 (16.9%) |  |
| **DDWR with intermittent locking (DC/TMD)** |  |  |  |  |  |  |
| No | 56 (83.6%) | 45 (76.3%) | 0,305 | 39 (90.7%)* | 62 (74.7%) | ***0,033*** |
| Yes | 11 (16.4%) | 14 (23.7%) |  | 4 (9.3%) | 21 (25.3%)* |  |
| **Orofacial pain (VAS)** | 3.74±2.36 | 2.54±2.33 | ***0,005*** | 3.37±2.26 | 3.08±2.50 | 0,516 |
| Up to 3 | 29 (43.3%) | 34 (57.6%) | 0,108 | 20 (46.5%) | 43 (51.8%) | 0,573 |
| Over 3 | 38 (56.7%) | 25 (42.4%) |  | 23 (53.5%) | 40 (48.2%) |  |
| **Orofacial fatigue (VAS)** | 3.40±2.19 | 2.76±2.18 | 0,102 | 2.99±2.26 | 3.16±2.18 | 0,692 |
| Up to 3 | 31 (46.3%) | 39 (66.1%)* | ***0,025*** | 24 (55.8%) | 46 (55.4%) | 0,966 |
| Over 3 | 36 (53.7%)* | 20 (33.9%) |  | 19 (44.2%) | 37 (44.6%) |  |
| **Orofacial Stiffness (VAS)** | 2.56±2.61 | 2.52±2.76 | 0,926 | 2.03±2.25 | 2.80±2.84 | 0,128 |
| Up to 2 | 33 (49.3%) | 31 (52.5%) | 0,713 | 23 (53.5%) | 41 (49.4%) | 0,663 |
| Over 2 | 34 (50.7%) | 28 (47.5%) |  | 20 (46.5%) | 42 (50.6%) |  |
| **Orofacial Stiffness (VAS)** | 0.91±1.91 | 0.79±1.86 | 0,739 | 0.77±1.85 | 0.90±1.91 | 0,702 |
| Up to 1 | 52 (77.6%) | 48 (81.4%) | 0,604 | 35 (81.4%) | 65 (78.3%) | 0,685 |
| Over 1 | 15 (22.4%) | 11 (18.6%) |  | 8 (18.6%) | 18 (21.7%) |  |
| **Orofacial joint instability** **(VAS)** | 3.20±3.48 | 3.34±3.17 | 0,818 | 2.76±3.38 | 3.53±3.29 | 0,219 |
| Up to 3 | 38 (56.7%) | 29 (49.2%) | 0,396 | 27 (62.8%) | 40 (48.2%) | 0,119 |
| Over 3 | 29 (43.3%) | 30 (50.8%) |  | 16 (37.2%) | 43 (51.8%) |  |
| **Generalized joint hypermobility** |  |  |  |  |  |  |
| No | 31 (46.3%) | 24 (40.7%) | 0,528 | 20 (46.5%) | 35 (42.2%) | 0,641 |
| Yes | 36 (53.7%) | 35 (59.3%) |  | 23 (53.5%) | 48 (57.8%) |  |
| **Right TMJ PPT (kgf)** | 1.11±0.45 | 1.40±0.54 | ***0,002*** | 1.05±0.41 | 1.35±0.53 | ***0,002*** |
| Up to 1,1 | 42 (62.7%)* | 20 (33.9%) | ***0,001*** | 32 (74.4%)* | 30 (36.1%) | ***<0,001*** |
| Over 1,1 | 25 (37.3%) | 39 (66.1%)* |  | 11 (25.6%) | 53 (63.9%)* |  |
| **Left TMJ PPT (kgf)** | 1.02±0.32 | 1.24±0.44 | ***0,001*** | 1.00±0.32 | 1.19±0.42 | ***0,012*** |
| Up to 1,1 | 45 (67.2%)* | 23 (39.0%) | ***0,002*** | 31 (72.1%)* | 37 (44.6%) | ***0,003*** |
| Over 1,1 | 22 (32.8%) | 36 (61.0%)* |  | 12 (27.9%) | 46 (55.4%)* |  |
| **Right masseter PPT (kgf)** | 1.20±0.46 | 1.45±0.57 | ***0,006*** | 1.19±0.46 | 1.38±0.55 | ***0,047*** |
| Up to 1,3 | 40 (59.7%)* | 22 (37.3%) | ***0,012*** | 28 (65.1%)* | 34 (41.0%) | ***0,010*** |
| Over 1,3 | 27 (40.3%) | 37 (62.7%)* |  | 15 (34.9%) | 49 (59.0%)* |  |
| **Left masseter PPT (kgf)** | 1.19±0.41 | 1.39±0.48 | ***0,015*** | 1.17±0.40 | 1.35±0.47 | ***0,037*** |
| Up to 1,3 | 43 (64.2%)* | 25 (42.4%) | ***0,014*** | 28 (65.1%) | 40 (48.2%) | 0,071 |
| Over 1,3 | 24 (35.8%) | 34 (57.6%)* |  | 15 (34.9%) | 43 (51.8%) |  |
| **Right temporalis PPT (kgf)** | 1.42±0.53 | 1.65±0.62 | ***0,026*** | 1.38±0.53 | 1.60±0.60 | ***0,036*** |
| Up to 1,3 | 33 (49.3%) | 20 (33.9%) | 0,081 | 23 (53.5%) | 30 (36.1%) | 0,062 |
| Over 1,3 | 34 (50.7%) | 39 (66.1%) |  | 20 (46.5%) | 53 (63.9%) |  |
| **Left temporalis PPT (kgf)** | 1.29±0.43 | 1.46±0.49 | ***0,045*** | 1.30±0.43 | 1.40±0.48 | 0,233 |
| Up to 1,3 | 40 (59.7%)* | 24 (40.7%) | ***0,033*** | 24 (55.8%) | 40 (48.2%) | 0,417 |
| Over 1,3 | 27 (40.3%) | 35 (59.3%)* |  | 19 (44.2%) | 43 (51.8%) |  |
| **Pre-fatigue MBF** | 44.81±14.50 | 54.65±18.05 | ***0,001*** | 45.14±15.64 | 51.63±17.23 | ***0,041*** |
| Up to 45 | 39 (58.2%)* | 19 (32.2%) | ***0,003*** | 25 (58.1%)* | 33 (39.8%) | ***0,049*** |
| Over 45 | 28 (41.8%) | 40 (67.8%)* |  | 18 (41.9%) | 50 (60.2%)* |  |
| **Endurance time** | 113.53±59.82 | 109.97±44.15 | 0,708 | 121.14±71.85 | 107.06±39.38 | 0,157 |
| Up to 100 | 29 (43.3%) | 32 (54.2%) | 0,220 | 17 (39.5%) | 44 (53.0%) | 0,151 |
| Over 100 | 38 (56.7%) | 27 (45.8%) |  | 26 (60.5%) | 39 (47.0%) |  |
| **Post- fatigue MBF** | 36.73±13.66 | 46.28±16.81 | ***0,001*** | 37.02±15.55 | 43.37±15.72 | ***0,033*** |
| Up to 40 | 43 (64.2%)* | 21 (35.6%) | ***0,001*** | 26 (60.5%) | 38 (45.8%) | 0,118 |
| Over 40 | 24 (35.8%) | 38 (64.4%)* |  | 17 (39.5%) | 45 (54.2%) |  |
| **Percentage change in MBF** | 37.55±18.63 | 46.39±20.20 | ***0,012*** | 36.66±19.63 | 44.29±19.50 | ***0,040*** |
| Up to 40 | 41 (61.2%) | 26 (44.1%) | 0,055 | 26 (60.5%) | 41 (49.4%) | 0,238 |
| Over 40 | 26 (38.8%) | 33 (55.9%) |  | 17 (39.5%) | 42 (50.6%) |  |
| **Subjective fatigue (VAS) (post-fatigue)** | 6.89±2.49 | 6.78±2.49 | 0,808 | 6.52±2.68 | 7.00±2.38 | 0,314 |
| Up to 7 | 28 (41.8%) | 27 (45.8%) | 0,654 | 20 (46.5%) | 35 (42.2%) | 0,641 |
| Over 7 | 39 (58.2%) | 32 (54.2%) |  | 23 (53.5%) | 48 (57.8%) |  |
| **Left TMJ articular capsule** | 1.63±1.71 | 1.46±0.68 | 0,477 | 1.35±0.53 | 1.66±1.59 | 0,225 |
| Up to 1,4 | 30 (44.8%) | 33 (55.9%) | 0,211 | 23 (53.5%) | 40 (48.2%) | 0,573 |
| Over 1,4 | 37 (55.2%) | 26 (44.1%) |  | 20 (46.5%) | 43 (51.8%) |  |
| **Right TMJ articular capsule** | 1.44±0.51 | 1.33±0.60 | 0,302 | 1.42±0.51 | 1.37±0.58 | 0,599 |
| Up to 1,4 | 27 (40.3%) | 36 (61.0%)* | ***0,020*** | 19 (44.2%) | 44 (53.0%) | 0,347 |
| Over 1,4 | 40 (59.7%)* | 23 (39.0%) |  | 24 (55.8%) | 39 (47.0%) |  |
| **Right masseter (rest)** | 12.96±2.38 | 12.89±2.49 | 0,866 | 12.90±2.46 | 12.95±2.42 | 0,924 |
| Up to 13 | 35 (52.2%) | 31 (52.5%) | 0,973 | 23 (53.5%) | 43 (51.8%) | 0,858 |
| Over 13 | 32 (47.8%) | 28 (47.5%) |  | 20 (46.5%) | 40 (48.2%) |  |
| **Right masseter (contraction)** | 14.83±2.50 | 14.90±2.59 | 0,878 | 14.68±2.63 | 14.96±2.49 | 0,563 |
| Up to 15 | 40 (59.7%) | 34 (57.6%) | 0,813 | 28 (65.1%) | 46 (55.4%) | 0,295 |
| Over 15 | 27 (40.3%) | 25 (42.4%) |  | 15 (34.9%) | 37 (44.6%) |  |
| **Left masseter (rest)** | 12.96±2.14 | 12.88±2.36 | 0,846 | 12.79±2.02 | 12.99±2.34 | 0,624 |
| Up to 13 | 36 (53.7%) | 28 (47.5%) | 0,482 | 23 (53.5%) | 41 (49.4%) | 0,663 |
| Over 13 | 31 (46.3%) | 31 (52.5%) |  | 20 (46.5%) | 42 (50.6%) |  |
| **Left masseter (contraction)** | 14.67±2.22 | 15.09±2.52 | 0,322 | 14.49±2.22 | 15.06±2.43 | 0,202 |
| Up to 15 | 41 (61.2%) | 28 (47.5%) | 0,122 | 27 (62.8%) | 42 (50.6%) | 0,192 |
| Over 15 | 26 (38.8%) | 31 (52.5%) |  | 16 (37.2%) | 41 (49.4%) |  |
| **Helplessness** | 6.36±4.23 | 5.76±3.69 | 0,405 | 6.28±4.11 | 5.98±3.94 | 0,687 |
| Up to 6 | 35 (52.2%) | 31 (52.5%) | 0,973 | 22 (51.2%) | 44 (53.0%) | 0,844 |
| Over 6 | 32 (47.8%) | 28 (47.5%) |  | 21 (48.8%) | 39 (47.0%) |  |
| **Magnification** | 4.66±3.20 | 3.95±2.54 | 0,176 | 4.67±3.35 | 4.14±2.67 | 0,336 |
| Up to 5 | 41 (61.2%) | 43 (72.9%) | 0,165 | 27 (62.8%) | 57 (68.7%) | 0,506 |
| Over 5 | 26 (38.8%) | 16 (27.1%) |  | 16 (37.2%) | 26 (31.3%) |  |
| **Rumination** | 5.82±4.74 | 5.08±3.81 | 0,342 | 5.77±4.68 | 5.33±4.15 | 0,588 |
| Up to 5 | 37 (55.2%) | 33 (55.9%) | 0,936 | 24 (55.8%) | 46 (55.4%) | 0,966 |
| Over 5 | 30 (44.8%) | 26 (44.1%) |  | 19 (44.2%) | 37 (44.6%) |  |
| **Total catastrophizing (0–52)** | 16.79±11.42 | 14.63±8.98 | 0,244 | 16.65±11.34 | 15.33±9.86 | 0,498 |
| Up to 15 | 28 (41.8%) | 34 (57.6%) | 0,076 | 18 (41.9%) | 44 (53.0%) | 0,235 |
| Over 15 | 39 (58.2%) | 25 (42.4%) |  | 25 (58.1%) | 39 (47.0%) |  |
| **Mandibular kinesiophobia** | 28.03±6.44 | 27.66±5.48 | 0,732 | 27.56±6.44 | 28.01±5.78 | 0,688 |
| Up to 30 | 41 (61.2%) | 38 (64.4%) | 0,710 | 28 (65.1%) | 51 (61.4%) | 0,686 |
| Over 30 | 26 (38.8%) | 21 (35.6%) |  | 15 (34.9%) | 32 (38.6%) |  |
| **Hypervigilance** | 45.04±15.06 | 42.73±13.72 | 0,371 | 45.00±15.34 | 43.42±14.01 | 0,563 |
| Up to 40 | 25 (37.3%) | 28 (47.5%) | 0,250 | 16 (37.2%) | 37 (44.6%) | 0,427 |
| Over 40 | 42 (62.7%) | 31 (52.5%) |  | 27 (62.8%) | 46 (55.4%) |  |
| **JFLS score** | 50.70±32.56 | 31.58±20.31 | ***<0,001*** | 45.86±32.37 | 39.61±27.10 | 0,254 |
| Up to 35 | 27 (40.3%) | 38 (64.4%)* | ***0,007*** | 19 (44.2%) | 46 (55.4%) | 0,231 |
| Over 35 | 40 (59.7%)* | 21 (35.6%) |  | 24 (55.8%) | 37 (44.6%) |  |
| **JFLS items 7 and 12** | 11.63±5.66 | 9.56±4.64 | ***0,028*** | 10.86±5.96 | 10.55±4.94 | 0,759 |
| Up to 10 | 27 (40.3%) | 35 (59.3%)* | ***0,033*** | 19 (44.2%) | 43 (51.8%) | 0,417 |
| Over 10 | 40 (59.7%)* | 24 (40.7%) |  | 24 (55.8%) | 40 (48.2%) |  |

Table 3. Bivariate analysis considering maximum assisted and unassisted mouth opening as predictors of clinical, functional, and psychosocial outcomes. *p<0.05, Fisher’s exact test or Pearson’s chi-square test (n, %); *p<0.05, Mann–Whitney test (mean ± SD).
