# Supplementary material for: Predictive Value of Diagnostic Methods for TMJ Hypermobility in the Associated Clinical and Functional Features of Temporomandibular Disorders: A Regression Study
Source: J Oral Rehabil. 2025 Nov 27;53(3):673–84. doi: 10.1111/joor.70120 (PMC12902195; doi:10.1111/joor.70120)
Supplement: Supplementary file 4 — Table S4: Bivariate analysis considering the presence of lateral condylar jump as a predictor of clinical, functional and psychosocial variables. *p < 0.05, Fisher's exact test or Pearson's chi‐square test (n, %); *p < 0.05, Mann–Whitney test (mean ± SD). [file JOOR-53-673-s002.docx]

|  | **Lateral condylar jump** | |  |
| --- | --- | --- | --- |
|  | **No** | **Yes** | **p-**  **value** |
| **Sex** |  |  |  |
| Male | 5 (20.0%) | 27 (26.7%) | 0,489 |
| Female | 20 (80.0%) | 74 (73.3%) |  |
| **Age** | 27.28±5.49 | 27.11±5.77 | 0,894 |
| Up to 25 | 12 (48.0%) | 46 (45.5%) | 0,825 |
| Over 25 | 13 (52.0%) | 55 (54.5%) |  |
| **Right TMJ angle** | 264.07±36.29 | 276.92±28.67 | 0,060 |
| Up to 280º | 15 (60.0%)* | 36 (35.6%) | ***0,026*** |
| Over 280º | 10 (40.0%) | 65 (64.4%)* |  |
| **Left TMJ angle** | 260.58±36.24 | 277.69±27.92 | ***0,011*** |
| Up to 280º | 18 (72.0%) | 37 (36.6%) | ***0,001*** |
| Over 280º | 7 (28.0%) | 64 (63.4%) |  |
| **Open-locking episodes** |  |  |  |
| Never | 20 (80.0%)* | 41 (40.6%) | ***0,005*** |
| Once in a lifetime | 3 (12.0%) | 25 (24.8%) |  |
| Once a year | 2 (8.0%) | 7 (6.9%) |  |
| Once a month | 0 (0.0%) | 14 (13.9%)* |  |
| More than once a month | 0 (0.0%) | 14 (13.9%)* |  |
| **Pain-free maximum mouth opening** | 39.40±11.53 | 42.83±12.28 | 0,208 |
| Up to 40 | 10 (40.0%) | 42 (41.6%) | 0,885 |
| Over 40 | 15 (60.0%) | 59 (58.4%) |  |
| **Unassisted maximum mouth opening** | 52.12±6.58 | 55.76±7.26 | ***0,024*** |
| Up to 55 | 19 (76.0%)* | 48 (47.5%) | ***0,011*** |
| Over 55 | 6 (24.0%) | 53 (52.5%)* |  |
| **Assisted maximum mouth opening** | 54.16±6.01 | 59.34±6.32 | ***<0,001*** |
| Up to 55 | 16 (64.0%)* | 27 (26.7%) | ***<0,001*** |
| Over 55 | 9 (36.0%) | 74 (73.3%)* |  |
| **Midline deviation during opening** |  |  |  |
| No | 10 (40.0%)* | 9 (8.9%) | ***<0,001*** |
| Yes | 15 (60.0%) | 92 (91.1%)* |  |
| **Terminal click** |  |  |  |
| No | 25 (100.0%)* | 85 (84.2%) | ***0,033*** |
| Yes | 0 (0.0%) | 16 (15.8%)* |  |
| **Subluxation diagnosis (DC/TMD)** |  |  |  |
| No | 25 (100.0%)* | 76 (75.2%) | ***0,005*** |
| Yes | 0 (0.0%) | 25 (24.8%)* |  |
| **Muscular TMD diagnosis (DC/TMD)** |  |  |  |
| None | 4 (16.0%) | 29 (28.7%) | 0,091 |
| Local myalgia | 10 (40.0%) | 18 (17.8%) |  |
| Myofascial pain | 7 (28.0%) | 28 (27.7%) |  |
| Myofascial pain with referral | 4 (16.0%) | 26 (25.7%) |  |
| **Headache attributed to TMD** |  |  |  |
| No | 12 (48.0%) | 56 (55.4%) | 0,504 |
| Yes | 13 (52.0%) | 45 (44.6%) |  |
| **Arthralgia** |  |  |  |
| No | 14 (56.0%) | 43 (42.6%) | 0,153 |
| Unilateral | 9 (36.0%) | 32 (31.7%) |  |
| Bilateral | 2 (8.0%) | 26 (25.7%) |  |
| **TMJ pain attributed to subluxation (ICOP)** |  |  |  |
| No | 25 (100.0%)* | 86 (85.1%) | ***0,040*** |
| Yes | 0 (0.0%) | 15 (14.9%)* |  |
| **Muscle pain diagnosis (ICOP)** |  |  |  |
| None | 4 (16.0%) | 31 (30.7%) | 0,324 |
| Acute primary orofacial myofascial pain | 1 (4.0%) | 2 (2.0%) |  |
| Frequent chronic primary orofacial myofascial pain without referred pain | 9 (36.0%) | 18 (17.8%) |  |
| Frequent chronic primary orofacial myofascial pain with referred pain | 2 (8.0%) | 6 (5.9%) |  |
| Highly frequent chronic primary orofacial myofascial pain without referred pain | 7 (28.0%) | 29 (28.7%) |  |
| Highly frequent chronic primary orofacial myofascial pain with referred pain | 2 (8.0%) | 15 (14.9%) |  |
| **TMJ pain diagnosis (ICOP)** |  |  |  |
| None | 18 (72.0%)* | 56 (55.4%) | ***0,023*** |
| Acute primary TMJ pain | 1 (4.0%) | 2 (2.0%) |  |
| Frequent chronic primary TMJ pain without referred pain | 1 (4.0%) | 10 (9.9%) |  |
| Frequent chronic primary TMJ pain with referred pain | 3 (12.0%) | 1 (1.0%) |  |
| Highly frequent chronic primary TMJ pain without referred pain | 0 (0.0%) | 7 (6.9%) |  |
| Highly frequent chronic primary TMJ pain with referred pain | 2 (8.0%) | 6 (5.9%) |  |
| MJ pain attributed to disc displacement with reduction | 0 (0.0%) | 12 (11.9%)* |  |
| TMJ pain attributed to subluxation | 0 (0.0%) | 7 (6.9%)* |  |
| **Disc displacement with reduction (DDWR) (DC/TMD)** |  |  |  |
| No | 9 (36.0%) | 25 (24.8%) | 0,134 |
| Unilateral | 15 (60.0%) | 56 (55.4%) |  |
| Bilateral | 1 (4.0%) | 20 (19.8%) |  |
| **DDWR with intermittent locking (DC/TMD)** |  |  |  |
| No | 24 (96.0%)* | 77 (76.2%) | ***0,027*** |
| Yes | 1 (4.0%) | 24 (23.8%)* |  |
| **Orofacial pain (VAS)** | 2.98±2.15 | 3.23±2.48 | 0,644 |
| Up to 3 | 12 (48.0%) | 51 (50.5%) | 0,823 |
| Over 3 | 13 (52.0%) | 50 (49.5%) |  |
| **Orofacial fatigue (VAS)** | 2.82±2.24 | 3.17±2.20 | 0,484 |
| Up to 3 | 16 (64.0%) | 54 (53.5%) | 0,343 |
| Over 3 | 9 (36.0%) | 47 (46.5%) |  |
| **Orofacial Stiffness (VAS)** | 1.35±2.23 | 2.83±2.70 | ***0,012*** |
| Up to 2 | 17 (68.0%) | 47 (46.5%) | 0,055 |
| Over 2 | 8 (32.0%) | 54 (53.5%) |  |
| **Orofacial Stiffness (VAS)** | 0.82±2.11 | 0.86±1.83 | 0,909 |
| Up to 1 | 21 (84.0%) | 79 (78.2%) | 0,522 |
| Over 1 | 4 (16.0%) | 22 (21.8%) |  |
| **Orofacial joint instability** **(VAS)** | 1.51±2.45 | 3.70±3.38 | ***0,003*** |
| Up to 3 | 20 (80.0%)* | 47 (46.5%) | ***0,003*** |
| Over 3 | 5 (20.0%) | 54 (53.5%)* |  |
| **Generalized joint hypermobility** |  |  |  |
| No | 13 (52.0%) | 42 (41.6%) | 0,347 |
| Yes | 12 (48.0%) | 59 (58.4%) |  |
| **Right TMJ PPT (kgf)** | 1.16±0.52 | 1.27±0.51 | 0,350 |
| Up to 1,1 | 16 (64.0%) | 46 (45.5%) | 0,098 |
| Over 1,1 | 9 (36.0%) | 55 (54.5%) |  |
| **Left TMJ PPT (kgf)** | 1.00±0.36 | 1.15±0.40 | 0,093 |
| Up to 1,1 | 19 (76.0%)* | 49 (48.5%) | ***0,014*** |
| Over 1,1 | 6 (24.0%) | 52 (51.5%)* |  |
| **Right masseter PPT (kgf)** | 1.16±0.48 | 1.36±0.54 | 0,106 |
| Up to 1,3 | 17 (68.0%)* | 45 (44.6%) | ***0,036*** |
| Over 1,3 | 8 (32.0%) | 56 (55.4%)* |  |
| **Left masseter PPT (kgf)** | 1.24±0.48 | 1.29±0.45 | 0,604 |
| Up to 1,3 | 14 (56.0%) | 54 (53.5%) | 0,820 |
| Over 1,3 | 11 (44.0%) | 47 (46.5%) |  |
| **Right temporalis PPT (kgf)** | 1.45±0.59 | 1.54±0.58 | 0,471 |
| Up to 1,3 | 11 (44.0%) | 42 (41.6%) | 0,827 |
| Over 1,3 | 14 (56.0%) | 59 (58.4%) |  |
| **Left temporalis PPT (kgf)** | 1.32±0.48 | 1.38±0.46 | 0,526 |
| Up to 1,3 | 14 (56.0%) | 50 (49.5%) | 0,561 |
| Over 1,3 | 11 (44.0%) | 51 (50.5%) |  |
| **Pre-fatigue MBF** | 51.08±19.43 | 49.00±16.33 | 0,586 |
| Up to 45 | 11 (44.0%) | 47 (46.5%) | 0,820 |
| Over 45 | 14 (56.0%) | 54 (53.5%) |  |
| **Endurance time** | 106.40±32.53 | 113.21±56.86 | 0,566 |
| Up to 100 | 11 (44.0%) | 50 (49.5%) | 0,622 |
| Over 100 | 14 (56.0%) | 51 (50.5%) |  |
| **Post- fatigue MBF** | 40.54±16.97 | 41.37±15.70 | 0,817 |
| Up to 40 | 12 (48.0%) | 52 (51.5%) | 0,755 |
| Over 40 | 13 (52.0%) | 49 (48.5%) |  |
| **Percentage change in MBF** | 41.58±19.27 | 41.71±20.03 | 0,977 |
| Up to 40 | 13 (52.0%) | 54 (53.5%) | 0,895 |
| Over 40 | 12 (48.0%) | 47 (46.5%) |  |
| **Subjective fatigue (VAS) (post-fatigue)** | 6.54±2.31 | 6.91±2.53 | 0,510 |
| Up to 7 | 15 (60.0%) | 40 (39.6%) | 0,066 |
| Over 7 | 10 (40.0%) | 61 (60.4%) |  |
| **Left TMJ articular capsule** | 1.38±0.51 | 1.60±1.46 | 0,475 |
| Up to 1,4 | 13 (52.0%) | 50 (49.5%) | 0,823 |
| Over 1,4 | 12 (48.0%) | 51 (50.5%) |  |
| **Right TMJ articular capsule** | 1.42±0.66 | 1.38±0.53 | 0,767 |
| Up to 1,4 | 14 (56.0%) | 49 (48.5%) | 0,503 |
| Over 1,4 | 11 (44.0%) | 52 (51.5%) |  |
| **Right masseter (rest)** | 13.43±1.82 | 12.81±2.54 | 0,253 |
| Up to 13 | 11 (44.0%) | 55 (54.5%) | 0,349 |
| Over 13 | 14 (56.0%) | 46 (45.5%) |  |
| **Right masseter (contraction)** | 15.27±2.21 | 14.76±2.61 | 0,371 |
| Up to 15 | 13 (52.0%) | 61 (60.4%) | 0,445 |
| Over 15 | 12 (48.0%) | 40 (39.6%) |  |
| **Left masseter (rest)** | 13.29±1.64 | 12.83±2.36 | 0,360 |
| Up to 13 | 10 (40.0%) | 54 (53.5%) | 0,228 |
| Over 13 | 15 (60.0%) | 47 (46.5%) |  |
| **Left masseter (contraction)** | 15.05±2.21 | 14.82±2.41 | 0,667 |
| Up to 15 | 13 (52.0%) | 56 (55.4%) | 0,757 |
| Over 15 | 12 (48.0%) | 45 (44.6%) |  |
| **Helplessness** | 5.08±4.12 | 6.33±3.93 | 0,162 |
| Up to 6 | 14 (56.0%) | 52 (51.5%) | 0,686 |
| Over 6 | 11 (44.0%) | 49 (48.5%) |  |
| **Magnification** | 3.64±3.25 | 4.50±2.82 | 0,191 |
| Up to 5 | 18 (72.0%) | 66 (65.3%) | 0,527 |
| Over 5 | 7 (28.0%) | 35 (34.7%) |  |
| **Rumination** | 4.52±4.72 | 5.71±4.21 | 0,218 |
| Up to 5 | 18 (72.0%) | 52 (51.5%) | 0,065 |
| Over 5 | 7 (28.0%) | 49 (48.5%) |  |
| **Total catastrophizing (0–52)** | 13.24±11.54 | 16.41±10.02 | 0,172 |
| Up to 15 | 16 (64.0%) | 46 (45.5%) | 0,098 |
| Over 15 | 9 (36.0%) | 55 (54.5%) |  |
| **Mandibular kinesiophobia** | 25.96±6.85 | 28.33±5.70 | 0,077 |
| Up to 30 | 17 (68.0%) | 62 (61.4%) | 0,540 |
| Over 30 | 8 (32.0%) | 39 (38.6%) |  |
| **Hypervigilance** | 46.00±14.86 | 43.46±14.36 | 0,432 |
| Up to 40 | 9 (36.0%) | 44 (43.6%) | 0,493 |
| Over 40 | 16 (64.0%) | 57 (56.4%) |  |
| **JFLS score** | 34.92±30.77 | 43.44±28.49 | 0,190 |
| Up to 35 | 15 (60.0%) | 50 (49.5%) | 0,347 |
| Over 35 | 10 (40.0%) | 51 (50.5%) |  |
| **JFLS items 7 and 12** | 8.20±5.22 | 11.27±5.15 | ***0,009*** |
| Up to 10 | 18 (72.0%)* | 44 (43.6%) | ***0,011*** |
| Over 10 | 7 (28.0%) | 57 (56.4%)* |  |

Table 4. Bivariate analysis considering the presence of lateral condylar jump as a predictor of clinical, functional, and psychosocial variables. *p<0.05, Fisher’s exact test or Pearson’s chi-square test (n, %); *p<0.05, Mann–Whitney test (mean ± SD).
